# Ranking of Nodal Infection Probability in Susceptible-Infected-Susceptible Epidemic

**DOI:** 10.1038/s41598-017-08611-9

**Published:** 2017-08-23

**Authors:** Bo Qu, Cong Li, Piet Van Mieghem, Huijuan Wang

**Affiliations:** 1 Faculty of Electrical Engineering, Mathematics and Computer Science, Delft University of Technology, Delft, 2628CD The Netherlands; 2 Adaptive Networks and Control Lab, Department of Electronic Engineering; and Research Center of Smart Networks and Systems, School of Information Science and Engineering, Fudan University, Shanghai, 200433 China

## Abstract

The prevalence, which is the average fraction of infected nodes, has been studied to evaluate the robustness of a network subject to the spread of epidemics. We explore the vulnerability (infection probability) of each node in the metastable state with a given effective infection rate *τ*. Specifically, we investigate the ranking of the nodal vulnerability subject to a susceptible-infected-susceptible epidemic, motivated by the fact that the ranking can be crucial for a network operator to assess which nodes are more vulnerable. Via both theoretical and numerical approaches, we unveil that the ranking of nodal vulnerability tends to change more significantly as *τ* varies when *τ* is smaller or in Barabási-Albert than Erdős-Rényi random graphs.

## Introduction

The continuous outbreaks of epidemic diseases in a population and viruses in computer networks^1–4^ motivate the study of epidemic spreading on a network. The Susceptible-Infected-Susceptible (SIS) epidemic process^5–12^ has been widely studied as a model of virus spread on a network. In the SIS model, a node is either infected or susceptible at any time *t*. Each infected node infects each of its susceptible neighbors with an infection rate *β*. Each infected node recovers with a recovery rate *δ*. Both infection and recovery processes are independent Poisson processes and the ratio *τ* = *β*/*δ* is the effective infection rate. There is an epidemic threshold *τ*_*c*_ and above the threshold *τ* > *τ*_*c*_ a nonzero fraction of nodes is infected in the metastable state. The infection probability *v*_*k*∞_(*τ*) of a node *k* in the metastable state at a given effective infection rate *τ* indicates the vulnerability of node *k* to the virus, and the prevalence, which equals the average fraction *y*_∞_(*τ*) of infected nodes reflects the global vulnerability of the network.

Researchers have mainly concentrated on the average fraction *y*_∞_ of infected nodes in the metastable state to estimate the vulnerability of a network against a certain epidemic or virus. Great effort has been devoted to understand how the network topology influences the vulnerability and the epidemic threshold^6, 13–15^, when the effective infection rate is just above the epidemic threshold [ref. 16, p. 469]. In this case, it is found [ref. 16, p. 469] that, the metastable-state infection probability vector (${V}_{\infty }={[{v}_{1\infty }{v}_{2\infty }\cdots {v}_{N\infty }]}^{T}$), obtained by the N-Intertwined Mean-Field Approximation (NIMFA) of SIS model is proportional to the principal eigenvector *x*_1_ of the adjacency matrix *A*. In this work, we aim to explore the nodal infection probability in a systematic way, in different network topologies and when the effective infection rate *τ* varies. As a starting point, we investigate the ranking of nodal infection probabilities, which crucially informs a network operator which nodes are more vulnerable or require protection. Interestingly, we find that the ranking of the nodal infection probability changes as the effective infection rate *τ* varies. The observation points out that we cannot find a topological feature of a node *i* to represent the vulnerability of node *i* to an SIS epidemic, because the rankings in vulnerability of nodes in a network may be different when the effective infection rate *τ* varies, whereas a topological feature of node *i* remains the same. Our observation explains the finding of Hebert-Dufresne *et al*.^17^ that different nodal features (such as degree, betweenness, *etc*.) should be used to select the nodes to immunize in different scenarios (based on different infection rates, link densities, *etc*.), *i.e*. different nodes should be immunized at different infection rates. In this paper, we explore two questions: (I) which network topology changes the ranking of nodal infection probabilities more significantly and (II) in which effective infection rate range, does the increment of the effective infection rate lead to a more significant change in the ranking for a given network topology?

We first assume that, for an arbitrary pair of nodes, the trajectory *v*_*k*∞_(*τ*) and *v*_*m*∞_(*τ*) as a function of the effective infection rate *τ* cross at most once in any interval (*τ*_0_, *τ*_1_). We call this assumption the one-crossing assumption and Section “Discussion about the one-crossing assumption” of the supplementary information shows that the assumption is reasonably good. Then the rankings of the vulnerabilities *v*_*k*∞_(*τ*) and *v*_*m*∞_(*τ*) change or equivalently the trajectories *v*_*k*∞_(*τ*) and *v*_*m*∞_(*τ*) cross if (*v*_*k*∞_(*τ*_0_) − *v*_*m*∞_(*τ*_0_) (*v*_*k*∞_(*τ*_1_) − *v*_*m*∞_(*τ*_1_) < 0, when the effective infection rate *τ* changes from *τ*_0_ to *τ*_1_. To estimate the maximal change in the ranking of nodal infection probabilities in a network, we consider the total number of crossings between the trajectories of the infection probabilities of all the nodes in a network, when the effective infection rate *τ* changes from just above the epidemic threshold to a large value, above which the ranking remains the same. The total number of crossings is a simple and straightforward measure of the changes in the ranking of nodal infection probabilities. (We also briefly discuss how the correlation of the ranking of nodal infection probabilities changes as the effective infection rate increases in Section “The Spearman rank correlation *ρ* as a function of *α*” of the supplementary information.) A higher total number of crossings may lead to a more complicated protection policy for a network operator. Given a network, we find a lower bound of the total number of crossings, which can be computed from the topology properties, in particular, from the degree vector and the principal eigenvector of the adjacency matrix. The lower bound is roughly proportional to, thus an accurate indicator of, the total number of crossings for an arbitrary network. Hence, these two topological features (*i.e*. the degree vector and the principal eigenvector of the adjacency matrix) could indeed characterize to what extent the ranking of nodal vulnerabilities would change on a network. Since the lower bound is computationally simple, it can be used to compare the total number of crossings for different network topologies. This result explains why the total number of crossings tends to be larger in networks with a smaller average degree if the degree distribution is given or with a larger degree variance if the average degree is given. Regarding to Question (II), we analytically derive the number of crossings when the effective infection rate *τ*_0_ increases with a small value *ε*, given the infection probability vector *V*_∞_(*τ*_0_) at the effective infection rate *τ*_0_. This theoretical result, validated by numerical results, explains the reason why the crossings are more likely to occur when the effective infection rate *τ* is smaller.

## Results

We first introduce in detail how to count or quantify the changes of the nodal ranking of the infection probability. Afterwards, we investigate the changes in the ranking (I) in different topologies when the effective infection rate *τ* increases from just above the epidemic threshold to a large value, above which the ranking remains the same, and (II) when the effective infection rate varies in different ranges.

### The counting of the nodal ranking changes

**Figure 1:**
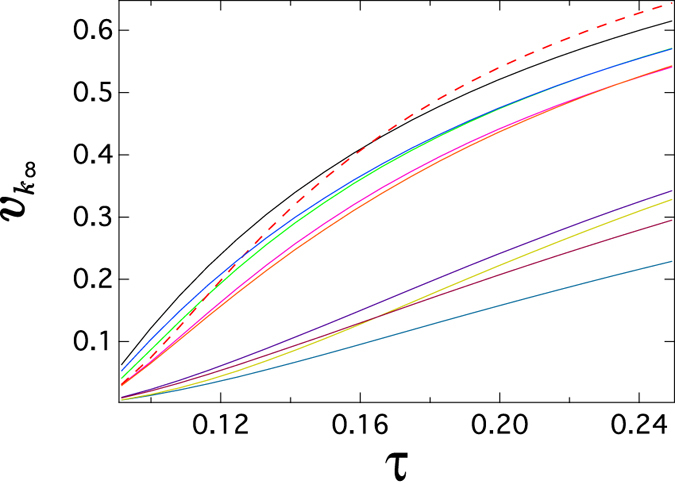
The meta-stable infection probabilit *v*_*k*∞_ as a function of the effective infection rate *τ* for 10 random nodes in a real-world network called Roget (details in Section “Real-world graph” of the supplementary information). The meta-stable infection probability *v*_*k*∞_ is obtained by solving (11).

To explore the changes of the nodal ranking of the infection probability, we investigate in a given network how many crossings, denoted by *χ*, between the trajectory *v*_*k*∞_(*τ*) and *v*_*m*∞_(*τ*) for all pairs of nodes can occur in the effective infection rate interval (*τ*_0_, *τ*_1_), where ${\tau }_{0} > {\tau }_{c}^{\mathrm{(1)}}$, and where ${\tau }_{c}^{\mathrm{(1)}}=\frac{1}{{\lambda }_{1}}$ (*λ*_1_ is the largest eigenvalue of the adjacency matrix) is the NIMFA epidemic threshold: the epidemic dies out if the effective infection rate $\tau  < {\tau }_{c}^{\mathrm{(1)}}$. (More details on ${\tau }_{c}^{\mathrm{(1)}}$ are introduced in Section “Methods”). In Fig. 1, we illustrate the trajectories *v*_*k*∞_(*τ*) of 10 nodes, randomly selected from a real-world network called Roget (*N* = 994 nodes, average degree *E* [*D*] = 7.32 and detailed in Section “Real-world graphs” of the supplementary information). For example, the vulnerability of the node corresponding to the red dash line in Fig. 1 changes dramatically from the medium vulnerability when *τ* = 0.12 to the high vulnerability when *τ* = 0.24. Network operators should be alert to such a change of nodal vulnerabilities. The trajectories *v*_*k*∞_(*τ*) of other groups of nodes in Roget are shown and discussed in the first section of the supplementary information.

For a graph with *N* nodes, the maximum possible number of crossings is $\frac{N(N-\mathrm{1)}}{2}$ under the one-crossing assumption. To count the number of crossings in the interval (*τ*_0_, *τ*_1_), we define an *N* × *N* matrix *F* with elements *f*_*ij*_:

$${f}_{ij}({V}_{\infty }({\tau }_{0}),{V}_{\infty }({\tau }_{1}))=({v}_{i\infty }({\tau }_{0})-{v}_{j\infty }({\tau }_{0}))\,({v}_{i\infty }({\tau }_{1})-{v}_{j\infty }({\tau }_{1}))$$

Since *f*_*ii*_ = 0, the matrix *F* has a zero diagonal just as the adjacency matrix *A*. A negative matrix element *f*_*ij*_ < 0 means that there is a crossing between the trajectory *v*_*i*∞_(*τ*) and *v*_*j*∞_(*τ*) in the interval (*τ*_0_, *τ*_1_). The number of crossings in the interval (*τ*_0_, *τ*_1_) of the effective infection rate then equals

1 $$\chi ({\tau }_{0},{\tau }_{1})=\sum _{i=1}^{N}\,\sum _{j=1}^{i-1}\,{1}_{{f}_{ij}({V}_{\infty }({\tau }_{0}),{V}_{\infty }({\tau }_{1})) < 0}$$

where 1_{*x*}_ is the indicator function: 1_{*x*}_ = 1 if the event or condition {*x*} is true, else 1_{*x*}_ = 0. Specifically, if all nodal degrees are the same in a random graph, the nodal ranking in any interval of *τ* does not change, since the infection probability of every node^6^ equals the average fraction of infected nodes for any effective infection rate *τ*. In this work, we focus on the NIMFA nodal infection probability in the meta-stable state which is obtained by solving (11), hence the initial conditions (such as how many nodes are initially infected) are not necessary.

We can compute the SIS metastable viral infection probability *v*_*k*∞_ of any node *k* both by the N-Intertwined Mean-Field Approximation (NIMFA)^6, 18^ and by simulations^8^ of the SIS continuous-time Markov process. We then further compare the number of crossings *χ* as a function of the increment in the effective infection rate *τ* over different ranges, obtained by NIMFA and the continuous-time simulations of the SIS model. As shown in Section “The comparison between NIFMA and the continuous-time simulation” of the supplementary information, the number of crossings obtained from NIMFA is relatively close to that from the simulations, so we compute the number *χ* of crossings mainly by NIMFA due to its computational efficiency. However, NIMFA may not be accurate when the effective infection rate is close to the epidemic threshold^8^. Hence, the number of crossings obtained by NIMFA and simulations may be different from each other when the effective infection rate is close to the epidemic threshold as shown in Section “The comparison between NIFMA and the continuous-time simulation” of the supplementary information.

### The total number of crossings in different topologies

We explore the total number of crossings in different graph topologies $\chi ({\tau }_{c}^{\mathrm{(1)}}+\varepsilon ,{\tau }_{u})$ when the effective infection rate *τ* changes from just above the epidemic threshold, *i.e*. ${\tau }_{c}^{\mathrm{(1)}}+\varepsilon$, to a large value *τ*_*u*_, above which the ranking of the nodal infection probability hardly changes. In Section “Methods – The derivation of the lower bound *χ*_*l*_”, we prove that there exists a value of *τ*, above which the ranking of the nodal infection probabilities does not change. We derive a lower bound of the total number of crossings and show that the lower bound is actually an accurate indicator of the total number of crossings in different types of graphs.

As shown in Section “Methods”, we derive a lower bound *χ*_*l*_ of the total number of crossings in a given graph:

2 $${\chi }_{l}=\sum _{i=1}^{N}\,\sum _{j=1}^{i-1}\,{1}_{{f}_{ij}({x}_{1},d) < 0}\le \chi ({\tau }_{c}^{\mathrm{(1)}}+\varepsilon ,{\tau }_{u})$$

where *x*_1_ is the principal eigenvector of the adjacency matrix *A*, belonging to the largest eigenvalue *λ*_1_ and *d* is the degree vector of the given graph.

**Figure 2:**
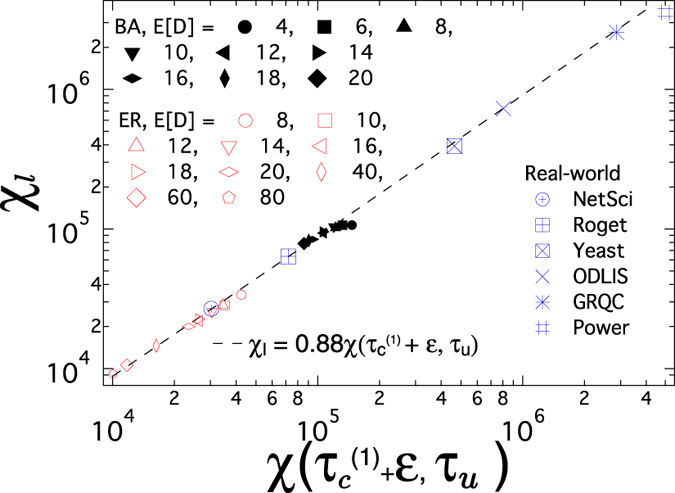
The lower bound *χ*_*l*_ versus the total number of crossings $\chi ({\tau }_{c}^{\mathrm{(1)}}+\varepsilon ,{\tau }_{u})$ in ER random graphs (with the size *N* = 1000), BA random graphs (with the size *N* = 1000) and real-world networks (details in Section “Real-world graphs” of the supplementary information).

With the one-crossing assumption, we can compute $\chi ({\tau }_{c}^{\mathrm{(1)}}+\varepsilon ,{\tau }_{u})$ from the infection probability vector ${V}_{\infty }({\tau }_{c}^{\mathrm{(1)}}+\varepsilon )$ and *V*_∞_(*τ*_*u*_). However, we have to select a proper value of *τ*_*u*_ which is large enough and practical. We set the value of *τ*_*u*_ as the minimum infection rate such that the average fraction of infected nodes *y*_∞_(*τ*_*u*_) ≥ 0.9, since we find for most Erdös-Rényi (ER), Barabási-Albert (BA) random graphs and the aforementioned real-world network, that the rankings of the nodal degree and the infection probability are almost the same when the average fraction of infected nodes *y*_∞_ ≥ 0.9. We discuss how we select the value of *τ*_*u*_ in Section “The value of *τ*_*u*_” of the supplementary information. The scatter plot of the lower bound *χ*_*l*_ vs versus $\chi ({\tau }_{c}^{\mathrm{(1)}}+\varepsilon ,{\tau }_{u})$ is shown in Fig. 2 for different graphs including ER random graphs, BA random graphs and six graphs constructed from real-world datasets (as described in Section “Real-world graphs” of the supplementary information), and the dash line in Fig. 2 is $\mathrm{log}\,{\chi }_{l}=\,\mathrm{log}\,\chi ({\tau }_{c}^{\mathrm{(1)}}+\varepsilon ,{\tau }_{u})+\,\mathrm{log}\,0.88$, equivalent to

3 $${\chi }_{l}=0.88\chi \,({\tau }_{c}^{\mathrm{(1)}}+\varepsilon ,{\tau }_{u})$$

We employ the average degree *E* [*D*] = 8, 10, 12, 14, 16, 18, 20, 40, 60, 80 for ER random graphs and *E* [*D*] = 4, 6, 8, 10, 12, 14, 16, 18, 20 for BA random graphs. Both ER and BA random graphs have the same size *N* = 1000. We confine ourselves to the connected graphs in this work. Hence, we employ the link density $p=\frac{{\rm{E}}[D]}{N-1}$ of ER random graphs, which is larger than the critical link density ${p}_{c}=\frac{\mathrm{ln}\,N}{N}\approx 0.007$ (equivalently the average degree E [*D*] > 7), to ensure the connectivity. Figure 2 and Equation (3) show that the lower bound *χ*_*l*_ is indeed always smaller than and approximately proportional to $\chi ({\tau }_{c}^{\mathrm{(1)}}+\varepsilon ,{\tau }_{u})$. Hence, the lower bound *χ*_*l*_ is a computationally simple indication of the total number of changes in the ranking of the metastable state infection probability in a graph. Moreover, we find that for graphs generated by the same random graph model (ER or BA model), a graph with a small average degree tends to have a large number of crossings; given the average degree, a graph with a large degree variance tends to have more crossings. We can understand this observation as follows. The principal eigenvector component of any node *i* obeys the eigenvalue equation ${({x}_{1})}_{i}={\sum }_{j=1}^{N}\,{a}_{ij}{({x}_{1})}_{j}$. The principal eigenvector is positively correlated with the degree vector^19^. Such correlation weakens if the principal eigenvector has a large variance, leading to a large *χ*_*l*_. When the degree variance is large, the variance of the principal eigenvector tends to be large as well, contributing to a large *χ*_*l*_. As more links are added to a network, the network becomes more homogeneous and the variance of the principal eigenvector decreases, resulting in a smaller *χ*_*l*_, or equivalently less crossings.

### The number of crossings in different intervals of *τ*

As shown in (1), we can compute the number *χ*(*τ*_0_, *τ*_1_) of crossings in the given interval (*τ*_0_, *τ*_1_) based on the knowledge of the infection probability vectors *V*_∞_(*τ*_0_) and *V*_∞_(*τ*_1_) only. Here, we show that we can theoretically derive the number of crossings in a small interval (*τ*_0_, *τ*_0_ + Δ*τ*) with the only knowledge of *V*_∞_(*τ*_0_). Afterwards, we will validate this theory by numerical results, and illustrate in which ranges of the effective infection rate the number of crossings tends to be larger.

#### The crossings close to a given *τ*

For sufficiently small *ε* = Δ*τ* > 0, the Taylor expansion of the steady-state NIMFA infection probability *v*_*k*∞_ for any node *k* is

4 $${v}_{k\infty }(\tau +\varepsilon )=\sum _{m=0}^{\infty }\,\frac{{\varepsilon }^{m}}{m!}\frac{{\partial }^{m}{v}_{k\infty }\,(\tau )}{\partial {\tau }^{m}}={v}_{k\infty }\,(\tau )+\varepsilon \frac{\partial {v}_{k\infty }\,(\tau )}{\partial \tau }+\frac{{\varepsilon }^{2}}{2}\frac{{\partial }^{2}{v}_{k\infty }\,(\tau )}{\partial {\tau }^{2}}+O\,({\varepsilon }^{3})$$

explicit up to order 2. In Section “Derivatives of *v*_*i*∞_ with respect to *τ*” of the supplementary information, we show the procedure to determine the *m*-th order derivative *v*_*i*∞_(*τ*) with respect to the effective infection rate *τ* for any node *k*.

If *v*_*k*∞_(*τ*) − *v*_*m*∞_(*τ*) > 0 and $\frac{\partial {v}_{k\infty }\,(\tau )}{\partial \tau }-\frac{\partial {v}_{m\infty }\,(\tau )}{\partial \tau } > 0$, then *v*_*k*∞_(*τ* + *ε*) − *v*_*m*∞_(*τ* + *ε*) > 0 for sufficiently small *ε* > 0 and the ranking at *τ* + *ε* and at *τ* is unchanged. On the other hand, if *v*_*k*∞_(*τ* + *ε*) − *v*_*m*∞_(*τ* + *ε*) = 0, which implies, for sufficiently small *ε* > 0 (so that we can ignore the higher order terms in *ε* ^*m*^ for *m* > 1 in (4)), that

$${v}_{k\infty }\,(\tau )-{v}_{m\infty }\,(\tau )\approx -\varepsilon \,(\frac{\partial {v}_{k\infty }\,(\tau )}{\partial \tau }-\frac{\partial {v}_{m\infty }\,(\tau )}{\partial \tau })$$

In other words, given *v*_*k*∞_(*τ*) of all nodes at *τ*, then there can be a zero or crossing at *τ* + *ε*_*km*_, where

5 $${\varepsilon }_{km}=-\frac{{v}_{k\infty }\,-{v}_{m\infty }\,(\tau )}{\frac{\partial {v}_{k\infty }\,(\tau )}{\partial \tau }-\frac{\partial {v}_{m\infty }\,(\tau )}{\partial \tau }}$$

if *ε*_*km*_ is small compared to *τ*. This approach is actually known as the Newton-Raphson method and corresponds with the first term in the Lagrange series for the inverse function (see ref. 20 in Page 304). A second order approximation, by ignoring terms of order *O*(*ε* ^3^) in (4), equating *v*_*k*∞_(*τ* + *ε*) − *v*_*m*∞_(*τ* + *ε*) = 0 and solving for *ε*, yields

6 $${\varepsilon }_{km}=\tfrac{-(\tfrac{\partial {v}_{k\infty }\,(\tau )}{\partial \tau }-\tfrac{\partial {v}_{m\infty }\,(\tau )}{\partial \tau })\pm \sqrt{{(\tfrac{\partial {v}_{k\infty }(\tau )}{\partial \tau }-\tfrac{\partial {v}_{m\infty }(\tau )}{\partial \tau })}^{2}-2(\tfrac{{\partial }^{2}{v}_{k\infty }\,(\tau )}{\partial {\tau }^{2}}-\tfrac{{\partial }^{2}{v}_{m\infty }\,(\tau )}{\partial {\tau }^{2}})\,({v}_{k\infty }\,(\tau )-{v}_{m\infty }\,(\tau ))}}{(\tfrac{{\partial }^{2}{v}_{k\infty }\,(\tau )}{\partial {\tau }^{2}}-\tfrac{{\partial }^{2}{v}_{m\infty }\,(\tau )}{\partial {\tau }^{2}})}$$

which is expected to be more accurate, in spite of the higher computational complexity since now also the set of second order derivatives needs to be solved. We rewrite (6) as

$${\varepsilon }_{km}=-(\tfrac{\tfrac{\partial {v}_{k\infty }\,(\tau )}{\partial \tau }-\tfrac{\partial {v}_{m\infty }\,(\tau )}{\partial \tau }}{\tfrac{{\partial }^{2}{v}_{k\infty }\,(\tau )}{\partial {\tau }^{2}}-\tfrac{{\partial }^{2}{v}_{m\infty }\,(\tau )}{\partial {\tau }^{2}}})\{1\pm \sqrt{1-2(\tfrac{\tfrac{{\partial }^{2}{v}_{k\infty }\,(\tau )}{\partial {\tau }^{2}}-\tfrac{{\partial }^{2}{v}_{m\infty }\,(\tau )}{\partial {\tau }^{2}}}{\tfrac{\partial {v}_{k\infty }\,(\tau )}{\partial \tau }-\tfrac{\partial {v}_{m\infty }\,(\tau )}{\partial \tau }})\,(\tfrac{{v}_{k\infty }\,(\tau )-{v}_{m\infty }\,(\tau )}{\tfrac{\partial {v}_{k\infty }\,(\tau )}{\partial \tau }-\tfrac{\partial {v}_{m\infty }\,(\tau )}{\partial \tau }})}\}$$

Using the generalized binomial expansion $${(1+x)}^{\alpha }={\sum }_{k=0}^{\infty }\,(\begin{array}{c}\alpha \\ k\end{array})\,{z}^{k}$$, valid for any |*z*| < 1, up to first order yields

$${\varepsilon }_{km}\simeq -(\tfrac{\tfrac{\partial {v}_{k\infty }\,(\tau )}{\partial \tau }-\tfrac{\partial {v}_{m\infty }\,(\tau )}{\partial \tau }}{\tfrac{{\partial }^{2}{v}_{k\infty }\,(\tau )}{\partial {\tau }^{2}}-\tfrac{{\partial }^{2}{v}_{m\infty }\,(\tau )}{\partial {\tau }^{2}}})\{1\pm [1-(\tfrac{\tfrac{{\partial }^{2}{v}_{k\infty }\,(\tau )}{\partial {\tau }^{2}}-\tfrac{{\partial }^{2}{v}_{m\infty }\,(\tau )}{\partial {\tau }^{2}}}{\tfrac{\partial {v}_{k\infty }\,(\tau )}{\partial \tau }-\tfrac{\partial {v}_{m\infty }\,(\tau )}{\partial \tau }})\,(\tfrac{{v}_{k\infty }\,(\tau )-{v}_{m\infty }\,(\tau )}{\tfrac{\partial {v}_{k\infty }\,(\tau )}{\partial \tau }-\tfrac{\partial {v}_{m\infty }\,(\tau )}{\partial \tau }})]\}$$

After only retaining the root with the minus sign, we arrive again at (5), illustrating that (5) is accurate when (5) is as small as possible (so that higher order evaluations are not needed). The discriminant must be positive in order to obtain feasible *ε*_*km*_. A positive discriminant is a condition for the existence of crossing in the interval (*τ*, *τ* + *ε*). Hence, given an effective infection rate *τ*_0_ and the corresponding infection probability vector *V*_∞_(*τ*_0_), there is a crossing close to *τ*_0_ between the trajectory *v*_*k*∞_(*τ*) and the trajectory *v*_*m*∞_(*τ*) at *τ* + *ε*_*km*_ if *ε*_*km*_ computed by (5) is positive and small enough.

#### Numerical results

In the following, we propose to normalize the effective infection rate by the NIMFA epidemic threshold: $\alpha =\frac{\tau }{{\tau }_{c}^{(1)}}\ge 1$, so that we can compare the number *χ* of crossings in different intervals of *α* in the same range (1, *α*_max_) for different network topologies, *i.e*. different average degrees and different degree distributions. We explore the crossings of the infection probability trajectories when the effective infection rate varies over the range (1, *α*_*max*_). We divide the range (1, *α*_max_) into intervals (*α*_*j*−1_, *α*_*j*_) where *j* = 1, 2, …, *n* is the index and *α*_*n*_ = *α*_*max*_.

**Figure 3:**
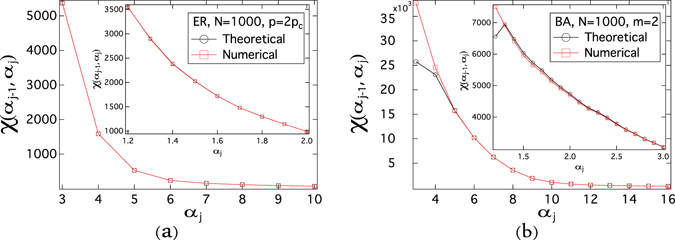
The number *χ*(*α*_*j*−1_, *α*_*j*_) of crossings as a function of the normalized effective infection rate *α*_*j*_. For ER graphs, we employ the link density *p* = 2*p*_*c*_, thus the average degree E [*D*] = 14, the size *N* = 1000 and the NIMFA epidemic threshold ${\tau }_{c}^{\mathrm{(1)}}\approx 0.0673$. For BA graphs, we employ the number of newly added links in each step *m* = 2, thus the average degree *E* [*D*] = 4, the size *N* = 1000, and the NIMFA epidemic threshold ${\tau }_{c}^{\mathrm{(1)}}\approx 0.0902$. The meta-stable infection probability *v*_*k*∞_ is obtained by solving (11) and the number *χ*(*α*_*j*−1_, *α*_*j*_) of crossings is obtained by (1). The results are averaged over 10 realizations.

We aim to explore in which interval of the normalized effective infection rate *α* the crossings are more likely to appear. Hence, instead of directly exploring the number of crossings between the trajectory of every node in the whole interval (1, *α*_max_) of the effective infection rate *α*, we investigate the number *χ*(*α*_*j*−1_, *α*_*j*_) of crossings in (1) in each small interval (*α*_*j*−1_, *α*_*j*_). We denote *α*_0_ = 1 (since the effective infection rate below the epidemic threshold corresponds to the all-healthy state), *α*_*n*_ = *α*_max_ and *α*_*j*_ = *α*_0_ + *j*Δ*α*, where Δ*α* = (*α*_max_ − 1)/*n* is the length of each interval. We will study how the number of crossings changes at different regions of the effective infection rate *τ* or scaled *α*. The infection probability *v*_*k*∞_(*α*) at any given value of the normalized effective infection rate *α* is computed by solving the NIMFA equation (11). On one hand, we can further compute the number *χ*(*α*_*j*−1_, *α*_*j*_) of crossings between all node pairs within any interval (*α*_*j*−1_, *α*_*j*_) by employing our theoretical result (5). On the other hand, we can also numerically compute the number *χ*(*α*_*j*−1_, *α*_*j*_) by (1). We first compare the theoretical (5) and numerical (1) when the normalized effective infection rate *α* is not close to 1, *i.e*. when the effective infection rate *τ* is not close to the epidemic threshold *τ*_*c*_; specifically, we start from *α*_0_ = 2 and *α*_*j*_ = *α*_0_ + *j*Δ*α*, where Δ*α* = 1. The main figures in Fig. 3 demonstrate that, for both ER and BA graphs, our theoretical result (5) agrees well with the numerical result (1) except for BA graphs in the interval (2, 3). The lower accuracy of our theoretical result for small *α* can be explained as follows. Compared to ${\tau }_{j-1}={\alpha }_{j-1}{\tau }_{c}^{\mathrm{(1)}}$, a small value of $({\alpha }_{j}-{\alpha }_{j-1})\,{\tau }_{c}^{\mathrm{(1)}}$ is required for the accuracy of the theoretical results (5), since *ε* in (5) is required to be small with respect to the given effective infection rate *τ*. Hence, when *α*_*j*_ is smaller, a smaller value of $({\alpha }_{j}-{\alpha }_{j-1})\,{\tau }_{c}^{\mathrm{(1)}}$ is needed for (5) to be accurate.

We further plot the number *χ*(*α*_*j*−1_, *α*_*j*_) of crossings in the interval (*α*_*j*−1_, *α*_*j*_) as a function of *α*_*j*_, when the normalized effective infection rate *α* is close to 1 and the length of the interval is reduced to Δ*α* = 0.1. When the length of the interval, *i.e*. Δ*α*, is smaller, the theoretical (5) results are more consistent with the numerical (1) results for BA random graphs in the range of *α* ∈ (2, 3) in the inset than in the main figure of Fig. 3(b). For both ER and BA graphs, the two methods agree with each other well when the intervals of *α* are small, even when the normalized effective infection rate *α* is close to 1 as shown in the insets of Fig. 3.

#### Physical explanation

Figure 3 shows that more crossings appear when the effective infection rate is smaller. In this section, we give a physical explanation of that observation.

At an effective infection rate *τ* or a normalized effective infection rate *α*, Equation (14) shows that the comparison of the infection probabilities *v*_*k*∞_(*α*) and *v*_*m*∞_(*α*) is actually equivalent to the comparison of the sum of the infection probabilities of their neighbors, *i.e*. ${\sum }_{j\mathrm{=1}}^{N}\,{a}_{kj}{v}_{j\infty }(\alpha )$ and ${\sum }_{j\mathrm{=1}}^{N}{a}_{mj}{v}_{j\infty }(\alpha )$. Without loss of generality, we assume that the degree *d*_*k*_ of node *k* is larger than the degree *d*_*m*_ of node *m*, *i.e*. *d*_*k*_ > *d*_*m*_. As discussed in Section “Methods”, the infection probability *v*_*k*∞_(*α*) > *v*_*m*∞_(*α*) if the effective infection rate is large enough. If there exists a value of *α*_1_ at which ${\sum }_{j=1}^{N}\,{a}_{kj}{v}_{j\infty }({\alpha }_{1}) < {\sum }_{j=1}^{N}\,{a}_{mj}{v}_{j\infty }({\alpha }_{1})$ while *d*_*k*_ > *d*_*m*_, there must be a crossing between *v*_*k*∞_(*α*) and *v*_*m*∞_(*α*) in the interval (*α*_1_,∞). If the infection probabilities *v*_*j*∞_(*α*) (where *j* = 1, 2, …, *N*) of all nodes vary in a larger range with respect to the average infection probability $\frac{1}{N}{\sum }_{j=1}^{N}\,{v}_{j\infty }$, *i.e*. the average fraction *y*_∞_ of infected nodes, then there may be a higher probability that ${\sum }_{j=1}^{N}\,{a}_{kj}{v}_{j\infty }(\alpha ) < {\sum }_{j=1}^{N}\,{a}_{mj}{v}_{j\infty }(\alpha )$ and thus more crossings could be expected when the effective infection rate *τ* exceeds *α*_1_. This hypothesis further motivates us to study the normalized standard deviation of the nodal infection probability:

7 $${\sigma }^{\ast }(\alpha )=\frac{\sqrt{{\sum }_{i=1}^{N}{({v}_{i\infty }(\alpha )-{y}_{\infty }(\alpha ))}^{2}/N}}{{y}_{\infty }(\alpha )}$$

(where we define ${\sigma }^{\ast }(\alpha =\mathrm{1)}={\mathrm{lim}}_{\alpha \downarrow 1}\,{\sigma }^{\ast }(\alpha )$) and explore whether a larger difference $|{\sigma }^{\ast }({\alpha }_{j-1})-{\sigma }^{\ast }({\alpha }_{j})|$ of σ* would imply more crossings in the interval (*α*_*j*−1_ *, α*_*j*_).

**Figure 4:**
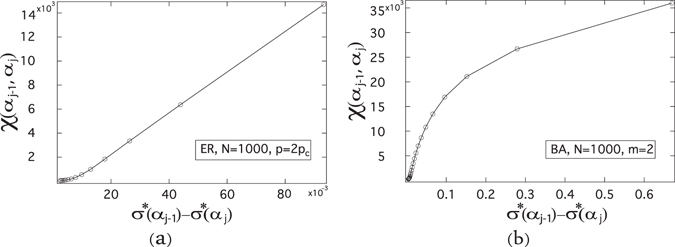
The number *χ*(*α*_*j*−1_, *α*_*j*_) of crossings as a function of the difference σ*(*α*_*j*−1_) − σ*(*α*_*j*_) of the normalized standard deviation of the metastable infection probability. For ER graphs, we employ the link density *p* = 2*p*_*c*_, thus the average degree E [*D*] = 14, and the size *N* = 1000 (the NIMFA epidemic threshold ${\tau }_{c}^{\mathrm{(1)}}\approx 0.0673$). For BA graphs, we employ the minimum degree *m* = 2, thus the average degree E [*D*] = 4, and the size *N* = 1000 (the NIMFA epidemic threshold ${\tau }_{c}^{\mathrm{(1)}}\approx 0.0902$). The meta-stable infection probability *v*_*k*∞_ is obtained by solving (11), the number *χ*(*α*_*j*−1_, *α*_*j*_) of crossings is obtained by (1) and the value of σ*(*α*) is obtained by (7). The results are averaged over 10 realizations.

The number *χ*(*α*_*j*−1_, *α*_*j*_) of crossings as a function of the difference σ*(*α*_*j*−1_) − σ*(*α*_*j*_) is shown in Fig. 4(a) for ER random graphs and in Fig. 4(b) for BA random graphs. For both ER and BA random graphs, the number *χ*(*α*_*j*−1_, *α*_*j*_) of crossings are positively correlated with the difference σ*(*α*_*j*−1_) − σ*(*α*_*j*_) in the interval (*α*_*j*−1_, *α*_*j*_). We observe the same in ER and BA random graphs with various average degrees though not shown here. The numerical results support that more crossings tend to appear in an interval where the variable σ* changes more.

**Figure 5:**
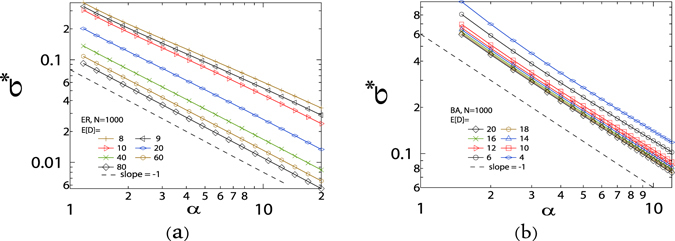
The normalized standard deviation σ* of infection probabilities of all nodes as a function of *α* in (**a**) ER and (**b**) BA random graphs. The dash line is a power-law curve with the exponent *γ* = −1. The sizes of all random graphs are 1000 and the average degree *E* [*D*] is shown in the figures. The meta-stable infection probability *v*_*k*∞_(*α*) is obtained by solving (11) and the value of σ*(*α*) is obtained by (7). The NIMFA epidemic threshold ${\tau }_{c}^{\mathrm{(1)}}\approx 0.1097$, 0.0993, 0.0902, 0.0476, 0.0244, 0.0164 and 0.0124 for ER random graphs with the average degree *E* [*D*] = 8, 9, 10, 20, 40, 60 and 80 respectively, and ${\tau }_{c}^{\mathrm{(1)}}\approx 0.0902$, 0.0698, 0.0479, 0.0416, 0.0368, 0.0329, 0.0300 and 0.0274 for BA random graphs with the average degree *E* [*D*] = 4, 6, 10, 12, 15, 16, 18 and 20 respectively. The results are averaged over 10 realizations.

We then further explore how the value of the variable σ*(*α*) changes with the normalized effective infection rate *α*. We plot the variable σ* as a function of the normalized effective infection rate *α* in Fig. 5(a) for ER random graphs and in Fig. 5(b) for BA random graphs with *N* = 1000 and various average degrees, and find that for both types of random graphs the curves can be fitted by a power law function, *i.e*. σ* is proportional to *α* ^−*γ*^, especially when the average degree is not small. More figures and the curve fittings are shown in the last section of the supplementary information for both ER and BA random graphs.

Figure 5 illustrates that the power law exponent *γ* of the fitting curves is close to 1 as the average degree E [*D*] increases for ER random graphs, and that is always approximately 1 for BA random graphs even though the average degree E [*D*] is small. Furthermore, the relationship between the variable σ* and the normalized effective infection rate *α* follows a power law when the effective infection rate is much larger, as shown in Section “σ* as a function of *τ*” of the supplementary information.

When *α* is large, we can theoretically prove the power-law relationship between the variable σ* and the normalized effective infection rate *α*. By (12) and assuming a large enough effective infection rate, we obtain ${v}_{i\infty }\,(\tau )=1-\frac{1}{\tau {d}_{i}}+O({\tau }^{-2})$ for node *i* and consequently ${y}_{\infty }\,(\tau )=1-\frac{1}{\tau }{\rm{E}}\,[\tfrac{1}{D}]+O({\tau }^{-2})$, so that (7) becomes

8 $${\sigma }^{\ast }=\frac{\sqrt{{\rm{Var}}\,[\tfrac{1}{D}]}}{\tau -{\rm{E}}\,[\tfrac{1}{D}]}+O({\tau }^{-2})$$

In a finite graph, Var $[\tfrac{1}{D}]$ and E $[\tfrac{1}{D}]$ are finite, hence σ* is proportional to *τ* ^−1^. The NIMFA epidemic threshold ${\tau }_{c}^{\mathrm{(1)}}$ is a constant for a given graph, and with ${\alpha }^{-1}={\tau }^{-1}{\tau }_{c}^{\mathrm{(1)}}$, we obtain that σ* is proportional to *α* ^−1^. Although the power-law relationship between σ* and *α* can be clearly observed in Fig. 5, the effective infection rate *τ* corresponding to the variable *α* in this figure may be smaller than 1 and the theoretical proof is only valid when the effective infection rate $\tau \gg 1$. Our result (8) is based on connected graphs, because the terms E $[\tfrac{1}{D}]$ and Var $[\tfrac{1}{D}]$ are undefined in unconnected graphs with isolated nodes.

The power-law decay of the variable σ* with the effective infection rate *τ* explains why there are more crossings when the effective infection rate is smaller.

#### Validation on a real-world network

**Figure 6:**
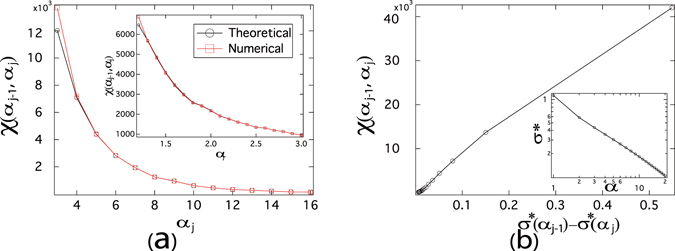
(**a**) The number *χ*(*α*_*j*−1_, *α*_*j*_) of crossings as a function of the normalized effective infection rate *α*_*j*_. (**b**) Main figure: the number *χ*(*α*_*j*−1_, *α*_*j*_) of crossings as a function of the difference σ*(*α*_*j*−1_) − σ*(*α*_*j*_) of the normalized standard deviation of the metastable infection probability; Inset: the normalized standard deviation σ* of infection probabilities of all nodes as a function of *α*. The real-world network – Roget, detailed in Section “Real-world graphs” of the supplementary information, is employed. The meta-stable infection probability *v*_*k*∞_ is obtained by solving (11), the number *χ*(*α*_*j*−1_, *α*_*j*_) of crossings is obtained by (1) and the value of σ*(*α*) is obtained by (7). The NIMFA epidemic threshold ${\tau }_{c}^{\mathrm{(1)}}\approx 0.0831$.

Finally, we validate our previous findings on the real-world network – Roget, detailed in Section “Real-world graphs” of the supplementary information. As shown in Fig. 6(a), the number *χ*(*α*_*j*−1_, *α*_*j*_) of crossings at normalized effective infection rate *α* interval obtained by theoretical and numerical methods are consistent with each other. The number of crossings decreases fast as *α* increases, similar to ER and BA models. The main figure of Fig. 6(b) shows that the number *χ*(*α*_*j*−1_, *α*_*j*_) of crossings increases with the difference σ*(*α*_*j*−1_) − σ*(*α*_*j*_) in the interval (*α*_*j*−1_, *α*_*j*_). In the inset of Fig. 6(b), we observe the power-law relationship between the variable σ* and the normalized effective infection rate *α*. All these findings are well in line with previous results on ER and BA random graphs.

## Discussion

In the SIS model, the infection probability trajectory *v*_*k*∞_(*τ*) of node *k* and the infection probability trajectory *v*_*m*∞_(*τ*) of node *m* may cross if (*v*_*k*∞_(*τ*_0_) − *v*_*m*∞_(*τ*_0_))(*v*_*k*∞_(*τ*_1_) − *v*_*m*∞_(*τ*_1_)) < 0, when the effective infection rate *τ* varies from *τ*_0_ to *τ*_1_. The number *χ*(*τ*_0_, *τ*_1_) of crossings of all node pairs within an interval (*τ*_0_, *τ*_1_) of the effective infection rate measures the change in the ranking of the nodal vulnerabilities when the effective infection rate changes from *τ*_0_ to *τ*_1_. We explore in what types of network topologies and in what ranges of the effective infection rates the crossings are more likely to appear. Theoretically, we find a lower bound *χ*_*l*_ in (2) of the total number of crossings in a graph. The lower bound *χ*_*l*_ only depends on topological features, *i.e*. the degree vector and principal eigenvector of the adjacency matrix. That lower bound *χ*_*l*_ is also shown to reflect the total number of crossings for a given graph. Moreover, we analytically predict the crossings close to an effective infection rate *τ*_0_, given the infection probabilities of all nodes at the effective infection rate *τ*_0_. This theory can be used to estimate the changes of the ranking of the nodal vulnerabilities if the effective infection rate *τ* slightly increases from its current value *τ*_0_. We find that more crossings tend to appear when the effective infection rate is smaller. Our findings may help network operators to estimate how significant the ranking of nodal vulnerabilities may change for a given change of the effective infection rate on a given network.

This work inspires interesting further questions. For example, how much is the change in the value of the nodal infection probabilities when the trajectories of the nodal infection probability crossing? Can we use the changes in the ranking of nodal infection probabilities to more effectively select the nodes to immunize?

## Methods

### Network construction

The Erdös-Rényi (ER) random graph^21^ is one of the most widely-used and well-studied models. In an ER random graph *G*_*p*_(*N*) with *N* nodes, each pair of nodes is connected with probability *p* independent from every other pair. The distribution of the degree of a random node is binomial: $${\rm{\Pr }}[D=k]=(\begin{array}{c}N-1\\ k\end{array})\,{p}^{k}{\mathrm{(1}-p)}^{N-1-k}$$ and the average degree E [*D*] = (*N* − 1)*p*. For large *N* and constant E [*D*], the degree distribution tends^16^ to a Poisson distribution: Pr [*D* = *k*] = *exp*(−*E* [*D*]) (E [*D*])^*k*^/*k*! Moreover, if the link density $p > {p}_{c}=\frac{\mathrm{ln}\,N}{N}$ for large *N*, the graph *G*_*p*_(*N*) is almost surely connected. We employ ER graphs with *p* = 2*p*_*c*_ (the average degree is approximately E [*D*] = 14) and *N* = 1000 as an example in some discussions, but consider the ER graphs with various average degrees when needed.

Besides the ER random graph, the scale-free model is often used to capture the scale-free degree distribution of the real-world networks such as the Internet^22^ and World Wide Web^23^. In this work, we consider the Barabási-Albert (BA) model^24^, which begins with an initial connected network of *m*_0_ nodes. At each step, a new node is connected to *m* ≤ *m*_0_ existing nodes. The probability that an existing node is chosen to be connected is proportional to the degree of the existing node. The degree distribution of BA random graphs^16^ is Pr [*D* = *k*] = *ck* ^−3^ for sufficiently large *N*, where $c={({\sum }_{k=m}^{N-1}{k}^{-3})}^{-1}$. The minimum degree of BA graphs is *m*, and we set *m*_0_ = *m* + 1 to generate a BA graph with *N* = 1000 nodes. Hence, the number of links is $L=\frac{{m}_{0}({m}_{0}-\mathrm{1)}}{2}+(N-{m}_{0})\,m=(N-\frac{{m}_{0}}{2})\,m$ and the average degree is E $[D]=\frac{2L}{N}=\frac{2N-{m}_{0}}{N}m$, thus approximately equals to 2*m*. We employ the BA random graphs with *m* = 2 (the average degree E [*D*] = 4) as an example in discussions and consider more average degrees when needed.

### The N-Intertwined Mean-Field Approximation of the SIS model

The N-Intertwined Mean-Field Approximation (NIMFA) is one of the most accurate approximation of the SIS model that takes into account the influence of the network topology^6^. The single governing equation for a node *i* in the NIMFA is

9 $$\frac{{\rm{d}}{v}_{i}(t)}{{\rm{d}}t}=-\delta {v}_{i}(t)+\beta \mathrm{(1}-{v}_{i}(t))\sum _{j=1}^{N}\,{a}_{ij}{v}_{j}(t)$$

where *v*_*i*_(*t*) is the infection probability of node *i* at time *t*, and the adjacency matrix element *a*_*ij*_ = 1 or 0 denotes if there is a link or not between node *i* and node *j*. With $V(t)={[{v}_{1}(t){v}_{2}(t)\cdots {v}_{N}(t)]}^{T}$, the matrix evolution equation of NIFMA is

10 $$\frac{{\rm{d}}V(t)}{{\rm{d}}t}=(\beta \,{\rm{diag}}\,\mathrm{(1}-{v}_{i}(t))A-\delta I)\,V(t)$$

where *A* is the *N* × *N* adjacency matrix of the network, *I* is the *N* × *N* identity matrix and diag (*v*_*i*_(*t*)) is the diagonal matrix with elements ${v}_{1}(t),{v}_{2}(t),\mathrm{....},{v}_{N}(t)$. In the steady state, defined by $\frac{{\rm{d}}V(t)}{{\rm{d}}t}=0$, or equivalently ${\mathrm{lim}}_{t\to \infty }\,{v}_{i}(t)={v}_{i\infty }$ and ${\mathrm{lim}}_{t\to \infty }\,V(t)={V}_{\infty }$, we have

11 $$(\tau \,{\rm{diag}}\,\mathrm{(1}-{v}_{i\infty })\,A-I)\,{V}_{\infty }=0$$

Given the network and the effective infection rate *τ*, we can numerically compute the infection probability *v*_*i*∞_ as a function of the effective infection rate *τ* for each node *i* by solving (11). The trivial, *i.e*. all-zero, solution indicates the absorbing state where all nodes are susceptible. The non-zero solution of *V*_∞_ in (11), if exists, points to the existence of a metastable state with a non-zero fraction of infected nodes. Or else, the metastable state can be figured as 0 or not existing. In this paper, we are interested in actually the metastable state.

Furthermore, the NIMFA epidemic threshold ${\tau }_{c}^{\mathrm{(1)}}=\frac{1}{{\lambda }_{1}}$, where *λ*_1_ is the largest eigenvalue of the adjacency matrix *A*, is a lower bound of the exact epidemic threshold *τ*_*c*_, *i.e*. ${\tau }_{c}^{\mathrm{(1)}} < {\tau }_{c}$. The epidemic dies out if the effective infection rate $\tau  < {\tau }_{c}^{\mathrm{(1)}}$. Since the NIMFA is the main approach in this work, we also employ the NIMFA epidemic threshold ${\tau }_{c}^{\mathrm{(1)}}$. The Laurent series of the steady-state infection probability is given by refs 16 and 25

12 $${v}_{i\infty }\,(\tau )=1+\sum _{m\mathrm{=1}}^{\infty }\,{\eta }_{m}\,(i)\,{\tau }^{-m}$$

possesses the coefficients ${\eta }_{1}\,(i)=-\frac{1}{{d}_{i}}$ and

13 $${\eta }_{2}\,(i)=\frac{1}{{d}_{i}^{2}}\,(1-\sum _{j=1}^{N}\,\frac{{a}_{ij}}{{d}_{j}})$$

and for *m* ≥ 2, the coefficients obey the recursion

$${\eta }_{m+1}\,(i)=-\frac{1}{{d}_{i}}\,{\eta }_{m}\,(i)\,(1-\sum _{j=1}^{N}\,\frac{{a}_{ij}}{{d}_{j}})-\frac{1}{{d}_{i}}\sum _{k=2}^{m}\,{\eta }_{m+1-k}\,(i)\,\sum _{j=1}^{N}\,{a}_{ij}{\eta }_{k}\,(j)$$

### The derivation of the lower bound *χ*_*l*_

As shown in [ref. 16, p. 469] when the effective infection rate $\tau ={\tau }_{c}^{\mathrm{(1)}}+\varepsilon$ is just above the NIMFA epidemic threshold ${\tau }_{c}^{(1)}=\frac{1}{{\lambda }_{1}}$, the vector *V*_∞_ with the NIMFA metastable-state infection probabilities is proportional to the principal eigenvector *x*_1_ of the adjacency matrix *A*. In particular, *v*_*k*∞_ = *ε*(*x*_1_)_*k*_, where *ε* > 0 and (*x*_1_)_*k*_ is the *k*-th component corresponding to node *k* of the principal eigenvector *x*_1_ of the adjacency matrix *A*, belonging to the largest eigenvalue *λ*_1_. The Perron-Frobenius Theorem^20^ states that all vector components of *x*_1_ are non-negative, and even positive if the graph *G* is connected. Hence, when the effective infection rate is just above the epidemic threshold, the ranking of the infection probability ${v}_{i\infty }\,({\tau }_{c}^{\mathrm{(1)}}+\varepsilon )$ is the same as the ranking of the component of the principal eigenvector (*x*_1_)_*i*_, *i.e*. ${f}_{km}\,({V}_{\infty }\,({\tau }_{c}^{\mathrm{(1)}}+\varepsilon ),{x}_{1})=0$ for any *k* and *m*.

On the other hand, the NIMFA steady-state infection probability for node *k* is given by ref. 18, [ref. 16, p. 464] and expressed as

14 $${v}_{k\infty }\,(\tau )=1-\frac{1}{1+\tau \,\sum _{j=1}^{N}\,{a}_{kj}{v}_{j\infty }(\tau )}$$

from which we obtain

$${v}_{k\infty }\,(\tau )-{v}_{m\infty }\,(\tau )=\tau \,(1-{v}_{k\infty }\,(\tau ))\,(1-{v}_{m\infty }\,(\tau ))\,\sum _{j=1}^{N}\,({a}_{kj}-{a}_{mj})\,{v}_{j\infty }\,(\tau )$$

The sign of *v*_*k*∞_(*τ*) − *v*_*m*∞_(*τ*) thus equals to the sign of ${\sum }_{j=1}^{N}\,({a}_{kj}-{a}_{mj})\,{v}_{j\infty }(\tau )$. Common neighbors of node *m* and *k* do not play a role in the sign change of *v*_*k*∞_(*τ*) − *v*_*m*∞_(*τ*). (The common neighbors of node *m* and *k* are the set of nodes $\{j\in {\mathscr{N}}\,:{a}_{mj}={a}_{kj}\}$). Moreover, if the number of non-common neighbors is 1 (or 0), then there is no change in the sign of *v*_*k*∞_(*τ*) − *v*_*m*∞_(*τ*) while the effective infection rate *τ* varies. Since the minimum infection probability *v*_min_(*τ*) > 0 for $\tau  > {\tau }_{c}^{(1)}$ as shown in [ref. 16, Lemma 17.4.2 on p. 464], the following bounds apply

$${d}_{k}{v}_{{\rm{\min }}}\,(\tau )-{d}_{m}{v}_{{\rm{\max }}}\,(\tau )\le \sum _{j=1}^{N}\,({a}_{kj}-{a}_{mj})\,{v}_{j\infty }\,(\tau )\le {d}_{k}{v}_{{\rm{\max }}}\,(\tau )-{d}_{m}{v}_{{\rm{\min }}}\,(\tau )$$

where *v*_max_(*τ*) and *v*_min_(*τ*) are the maximum and minimum infection probability respectively and *d*_*k*_ is the degree of node *k*, so that the condition *v*_*k*∞_(*τ*) − *v*_*m*∞_(*τ*) > 0 at *τ* is surely satisfied if ${d}_{k}-{d}_{m}\frac{{v}_{{\rm{\max }}}\,(\tau )}{{v}_{{\rm{\min }}(\tau )}} > 0$. Using ${v}_{{\rm{\max }}}\,(\tau )\le 1-\frac{1}{1+\tau {d}_{{\rm{\max }}}}$ and ${v}_{{\rm{\min }}}\,(\tau )\ge 1-\frac{1}{\tau {d}_{{\rm{\min }}}}$ in [ref. 16, p. 464–465], we arrive at the conservative bound for the condition *v*_*k*∞_(*τ*) − *v*_*m*∞_(*τ*) > 0 at *τ*,

$${d}_{k} > {d}_{m}\frac{{\tau }^{2}}{(\tau -\frac{1}{{d}_{{\rm{\min }}}})\,(\tau +\frac{1}{{d}_{{\rm{\max }}}})}$$

Hence, for large *τ*, the comparison between *v*_*k*∞_(*τ*) and *v*_*m*∞_(*τ*) reduces to a comparison in the nodal degree: if *d*_*k*_ > *d*_*m*_, then *v*_*k*∞_(*τ*) > *v*_*m*∞_(*τ*). This conclusion implies that there exists an effective infection rate *τ*_*u*_, above which the ranking of the metastable-state infection probability is the same as the ranking of the nodal degree, *i.e*. *f*_*km*_(*V*_∞_(*τ*), *d*) = 0 for any *k* and *m* (where *d* is the degree vector), if *τ* ≥ *τ*_*u*_.

The above discussion suggests that the number $\chi ({\tau }_{c}^{\mathrm{(1)}}+\varepsilon ,{\tau }_{u})$ of crossings in the interval $({\tau }_{c}^{\mathrm{(1)}}+\varepsilon ,{\tau }_{u})$ is the total number of crossings which a graph can possess. With the one-crossing assumption, we have

15 $$\chi ({\tau }_{c}^{\mathrm{(1)}}+\varepsilon ,{\tau }_{u})=\sum _{i=1}^{N}\,\sum _{j=1}^{i-1}\,{1}_{{f}_{ij}({V}_{\infty }({\tau }_{c}^{\mathrm{(1)}}+\varepsilon ),{V}_{\infty }({\tau }_{u})) < 0}\ge \sum _{i=1}^{N}\,\sum _{j=1}^{i-1}\,{1}_{{f}_{ij}({x}_{1},d) < 0}$$

Since only the crossings between two nodes with different degrees are considered in ${\sum }_{i=1}^{N}\,{\sum }_{j=1}^{i-1}\,{1}_{{f}_{ij}({x}_{1},d) < 0}$, we obtain a lower bound of the total number $\chi ({\tau }_{c}^{\mathrm{(1)}}+\varepsilon ,{\tau }_{u})$ of crossings. In order to simplify the notation, we denote the lower bound of the total number of crossings by ${\chi }_{l}={\sum }_{i=1}^{N}\,{\sum }_{j=1}^{i-1}\,{1}_{{f}_{ij}({x}_{1},d) < 0}$.

## Electronic supplementary material

Supplementary information
