# Supplementary material for: Ranking of Nodal Infection Probability in Susceptible-Infected-Susceptible Epidemic
Source: Sci Rep. 2017 Aug 23;7:9233. doi: 10.1038/s41598-017-08611-9 (PMC5569095; doi:10.1038/s41598-017-08611-9)
Supplement: Supplementary file 1 — Supplementary information [file 41598_2017_8611_MOESM1_ESM.pdf]

Supplementary Information: The nodal infection probability in Susceptible-Infected-Susceptible epidemic spreading

Bo Qu[1], Cong Li[2,*], Piet van Mieghem[1], and Huijuan Wang[1]

[1]Delft University of Technology, Delft, 2624CZ, The Netherlands
[2]Fudan University, Shanghai, postcode, China
[*]cong_li@fudan.edu.cn

June 26, 2017

The crossing behavior of the trajectories $v_{k\infty}$

In this section, we use a real-world network as an example to illustrate the crossing behavior by plotting the infection probability $v_{k\infty}$ as a function of the effective infection rate τ for a small number e.g. 10 nodes. The real-world network is called Roget (detailed in Section Real-world graphs), with 994 nodes and the average degree $\mathrm{E}[D] = 7.32$. If we plot all values of the infection probability $v_{k\infty}$ as a function of the effective infection rate τ for a network with hundreds of nodes, it would be difficult to tell which two curves actually cross. Hence, we sample 10 nodes, but according to different strategies to illustrate the crossing behavior. In Fig. S1(a), S1(b), S1(c) and S1(d), 10 nodes are randomly selected from all nodes; in Fig. S1(e), S1(f) and S1(g), 10 nodes are random selected from the nodes with degree $d = 4$, 5 and 6 respectively; Thus, the 10 nodes selected have the same degree in each of these three figures; in Fig. S1(h), the top 10 nodes with largest degrees are selected. We find that the crossing of a pair of nodes is indeed significant with respect to the value of their infection probabilities, when the nodes have quite different degrees, as shown in Fig. S1(a), S1(b) and S1(c) where the nodes are selected randomly. The crossing is less significant when the nodes have similar degrees as shown in Fig. S1(e), S1(f), S1(g) and S1(h). Since most real-world networks have a heavy tail degree distribution, significant crossing/change in infection probability for pairs of nodes is expected when the infection probability varies.

Discussion about the one-crossing assumption

We assume that the two trajectories $v_{k\infty}(\alpha)$ and $v_{m\infty}(\alpha)$ crosses at most once as the effective infection rate α changes. Although our theoretical result about the lower bound of the total number of crossings does not depend on this assumption, our method to compute the number of crossings does depend on such an assumption. Hence, we discuss whether the assumption is reasonably good.

Our simulation results so far show that more than one crossing seldom happen. For example, in a real-world network – Roget, only three pairs of nodes have two crossings in their infection probability trajectories in the infection-rate intervals we observed.

When we count numerically the number of crossings between two infection probability curves when α is changed from 1 to any large value α_{max}, we divide the interval $(1, \alpha_{max})$ into a number of m bins. If $m = 1$, we could find maximally one crossing by comparing the infection probability of the two nodes at $\alpha = 1$ and at $\alpha = \alpha_{max}$ respectively. As the number of bins increases, we may have the possibility to discover the multiple crossings if they exist. Hence, we explore further whether we observe few node pairs whose infection probability curves cross twice is due to the the fact that the bin size we chose is not small enough. Would it be possible that actually two crossings exist within

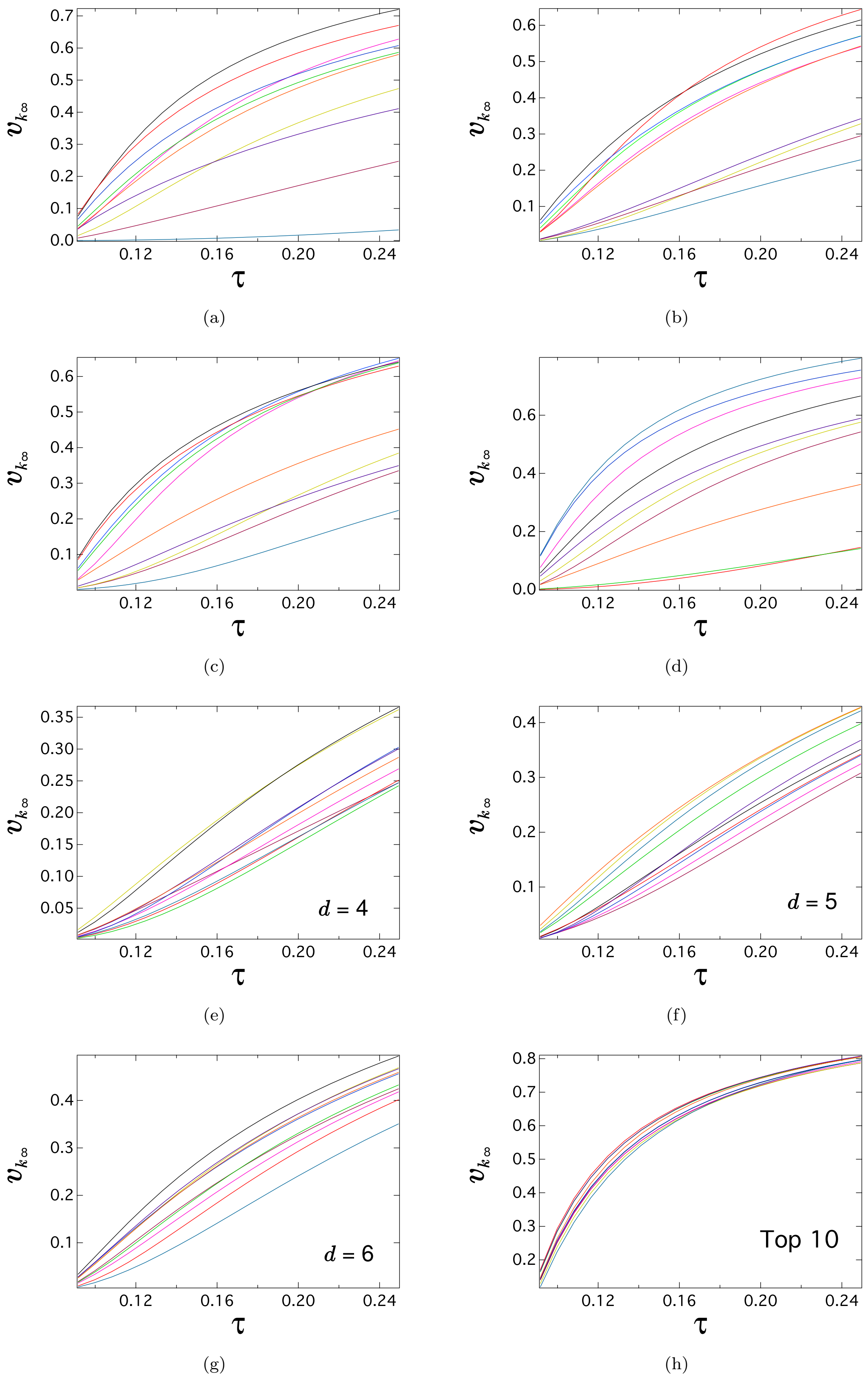

Figure S1: $v_{k\infty}$ as a function of τ for a real-world network.

the same bin which would not be observable if we don't split the bin into smaller ones. Hence, we gradually increase the number of bins to explore whether we could find more crossings. As shown in Fig. S2, we employ ER and BA random graphs with the average degree $\mathrm{E}[D] = 14$ as the examples to show how the number of crossings change when the interval is divided into small ones. We plot the number $\chi(1, \alpha)$ of crossings as a function of the normalized effective infection rate α. We do not observe evident increase of the number of crossings (taking all node pairs into account) as the number of bins increases.

Finally, the bin size should not be too small either. As the bin size becomes small, the change of infection probability for each node when the infection rate is changed from α to $\alpha + \epsilon$ is small. In this case, the precision of the numerical solution to compute the infection probability of each node using NIMFA may not be able to distinguish the ranking change of two nodes if their infection probabilities are close. The seemingly two crossings of a node pair may be due to the limited precision of our numerical solution when the bin size is too small.

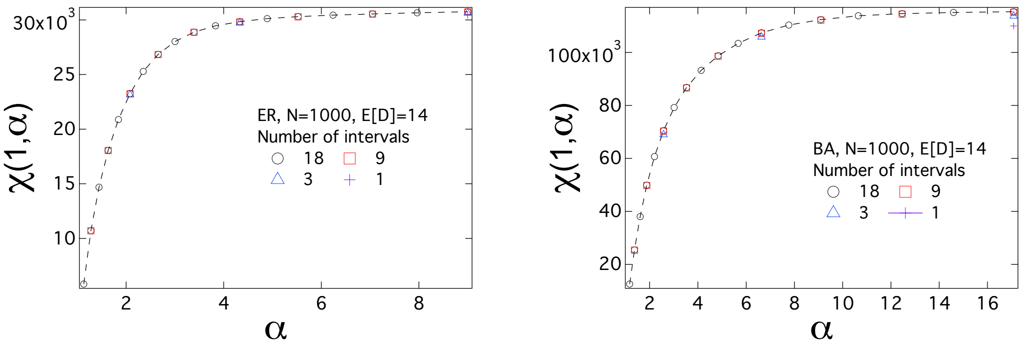

(a)

(b)

Figure S2: The number $\chi(1, \alpha)$ of crossings as a function of the normalized effective infection rate α for (a) ER random graphs and (b) BA random graphs with the same average degree $E[D] = 14$.

Derivatives of $v_{i\infty}$ with respect to τ

Only for vectors, we use the notation in which a function of a vector is equal to that function applied to the vector components; thus $f(R) = (f(r_1), f(r_2), \ldots, f(r_N))$. Obviously, this convention does not apply to matrices, where the matrix $f(A)$ is different than the matrix with elements $f(a_{ij})$.

Theorem 1 *Let V_∞ be the $N \times 1$ vector with k-th component $v_{k\infty}$, which obeys the NIMFA steady-state equation. Then, all higher order derivative vectors $\frac{\partial^m V_\infty}{\partial \tau^m}$ obey the linear equation*

$$\mathcal{Q}\left(\frac{1}{\tau(1-v_{i\infty})^2}\right)\frac{\partial^m V_\infty}{\partial \tau^m} = R_m \tag{1}$$

where $\mathcal{Q}(q_i) = diag(q_i) - A$ is the generalized Laplacian and where the right-hand side vector R_m depends on the previously computed vectors $\left(V_\infty, \frac{\partial V_\infty}{\partial \tau}, \ldots, \frac{\partial^{m-1} V_\infty}{\partial \tau^{m-1}}\right)$. In addition, the generalized Laplacian matrix $\mathcal{Q}\left(\frac{1}{\tau(1-v_{i\infty})^2}\right)$ and its inverse are positive definite matrices.

Proof: Following the approach in [1], the i-th component of the governing steady-state equation [1, (17.45) on p.466]

$$\frac{1}{\tau}\text{diag}\left(\frac{1}{1-v_{i\infty}}\right)V_\infty = AV_\infty \tag{2}$$

written as a generalized Laplacian $\mathcal{Q}(q_i) = \text{diag}(q_i) - A$,

$$\mathcal{Q}\left(\frac{1}{\tau(1-v_{i\infty})}\right)V_\infty = 0$$

is

$$\frac{v_{i\infty}}{1-v_{i\infty}} = \tau\sum_{j=1}^{N} a_{ij}v_{j\infty}$$

With $\frac{v_{i\infty}}{1-v_{i\infty}} = \frac{1}{1-v_{i\infty}} - 1$ and $\sum_{j=1}^{N} a_{kj}v_{j\infty} = \frac{v_{k\infty}}{\tau(1-v_{k\infty})}$, differentiation with respect to τ yields

$$\frac{1}{(1-v_{k\infty})^2}\frac{\partial v_{k\infty}(\tau)}{\partial \tau} - \tau\sum_{j=1}^{N} a_{kj}\frac{\partial v_{j\infty}(\tau)}{\partial \tau} = \sum_{j=1}^{N} a_{kj}v_{j\infty} = \frac{v_{k\infty}}{\tau(1-v_{k\infty})} \tag{3}$$

In matrix form, with the definition [1, p. 472] of the generalized Laplacian $\mathcal{Q}(q_i) = \text{diag}(q_i) - A$, the vector with the derivatives obeys

$$\mathcal{Q}\left(\frac{1}{\tau(1-v_{i\infty})^2}\right)\frac{\partial V_\infty}{\partial \tau} = \frac{1}{\tau^2}\text{diag}\left(\frac{1}{1-v_{i\infty}}\right)V_\infty = \frac{1}{\tau^2}\frac{V_\infty}{1-V_\infty} \tag{4}$$

In [2], we have shown that $\mathcal{Q}\left(\frac{1}{\tau(1-v_{i\infty})^2}\right)$ is positive definite (as well as its inverse),

$$\frac{\partial V_\infty}{\partial \tau} = \frac{1}{\tau^2}\mathcal{Q}^{-1}\left(\frac{1}{\tau(1-v_{i\infty})^2}\right)\text{diag}\left(\frac{1}{1-v_{i\infty}}\right)V_\infty$$

from which

$$\frac{\partial v_{k\infty}(\tau)}{\partial \tau} = \frac{1}{\tau^2}\sum_{j=1}^{N}\left(\mathcal{Q}^{-1}\left(\frac{1}{\tau(1-v_{i\infty})^2}\right)\right)_{kj}\frac{v_{j\infty}}{1-v_{j\infty}} \geq 0$$

Hence, given the knowledge of the vector V_∞ at the effective infection rate τ, the solution of a linear set returns the components of the vector $\frac{\partial V_\infty}{\partial \tau}$.

After a second differentiation with respect to τ of (3) and some manipulations, we have

$$\frac{1}{\tau\left(1-v_{k\infty}\right)^2}\frac{\partial^2 v_{k\infty}(\tau)}{\partial\tau^2}-\sum_{j=1}^{N}a_{kj}\frac{\partial^2 v_{j\infty}(\tau)}{\partial\tau^2}=\frac{2}{\tau}\sum_{j=1}^{N}a_{kj}\frac{\partial v_{j\infty}(\tau)}{\partial\tau}-\frac{2}{\tau\left(1-v_{k\infty}\right)^3}\left(\frac{\partial v_{k\infty}(\tau)}{\partial\tau}\right)^2$$

In matrix form, we obtain

$$\mathcal{Q}\left(\frac{1}{\tau\left(1-v_{i\infty}\right)^2}\right)\frac{\partial^2 V_\infty}{\partial\tau^2}=\frac{2}{\tau}A\frac{\partial V_\infty}{\partial\tau}-\frac{2}{\tau}\text{diag}\left(\frac{1}{\left(1-v_{k\infty}\right)^3}\right)\left(\frac{\partial V_{k\infty}(\tau)}{\partial\tau}\right)^2$$

We can avoid the matrix computation $A\frac{\partial V_\infty}{\partial\tau}$, because (4) supplies us with

$$\begin{aligned}A\frac{\partial V_\infty}{\partial\tau}&=\text{diag}\left(\frac{1}{\tau\left(1-v_{i\infty}\right)^2}\right)\frac{\partial V_\infty}{\partial\tau}-\frac{1}{\tau^2}\text{diag}\left(\frac{1}{1-v_{i\infty}}\right)V_\infty\\&=\frac{\frac{\partial V_\infty}{\partial\tau}}{\tau\left(1-V_\infty\right)^2}-\frac{1}{\tau^2}\frac{V_\infty}{1-V_\infty}\end{aligned}$$

while the NIMFA matrix equation (2) shows that

$$A\frac{\partial V_\infty}{\partial\tau}=\frac{d}{d\tau}\frac{1}{\tau}\frac{V_\infty}{1-V_\infty}=\frac{d}{d\tau}\frac{1}{\tau}\left(\frac{1}{1-V_\infty}-u\right)$$

where $u=(1,1,\ldots,1)$ is the all-one vector. Hence,

$$\mathcal{Q}\left(\frac{1}{\tau\left(1-v_{i\infty}\right)^2}\right)\frac{\partial^2 V_\infty}{\partial\tau^2}=R_2 \tag{5}$$

with

$$R_2=\frac{2}{\tau^2}\text{diag}\left(\frac{1}{\left(1-v_{i\infty}\right)^2}\right)\frac{\partial V_\infty}{\partial\tau}-\frac{2}{\tau}\text{diag}\left(\frac{1}{\left(1-v_{k\infty}\right)^3}\right)\left(\frac{\partial V_\infty(\tau)}{\partial\tau}\right)^2-\frac{2}{\tau^3}\text{diag}\left(\frac{1}{1-v_{i\infty}}\right)V_\infty$$

or

$$R_2=\frac{2}{\tau}\left\{\frac{d}{d\tau}\frac{1}{\tau}\left(\frac{1}{1-V_\infty}-u\right)-\frac{\left(\frac{\partial V_\infty(\tau)}{\partial\tau}\right)^2}{\left(1-V_\infty\right)^3}\right\}$$

which is a same matrix equation as in (4), but a different right-hand side vector, which can only be determined, after solving (4).

A next differentiation with respect to τ of

$$\frac{1}{\left(1-v_{k\infty}\right)^2}\frac{\partial^2 v_{k\infty}(\tau)}{\partial\tau^2}-\tau\sum_{j=1}^{N}a_{kj}\frac{\partial^2 v_{j\infty}(\tau)}{\partial\tau^2}=2\sum_{j=1}^{N}a_{kj}\frac{\partial v_{j\infty}(\tau)}{\partial\tau}-\frac{2}{\left(1-v_{k\infty}\right)^3}\left(\frac{\partial v_{k\infty}(\tau)}{\partial\tau}\right)^2$$

shows that the left-hand side L and the right-hand side R derivatives are

$$\begin{aligned}L&=\frac{1}{\left(1-v_{k\infty}\right)^2}\frac{\partial^3 v_{k\infty}(\tau)}{\partial\tau^3}+\frac{2}{\left(1-v_{k\infty}\right)^3}\frac{\partial^2 v_{k\infty}(\tau)}{\partial\tau^2}\frac{\partial v_{j\infty}(\tau)}{\partial\tau}-\sum_{j=1}^{N}a_{kj}\frac{\partial^2 v_{j\infty}(\tau)}{\partial\tau^2}-\tau\sum_{j=1}^{N}a_{kj}\frac{\partial^3 v_{j\infty}(\tau)}{\partial\tau^3}\\R&=2\sum_{j=1}^{N}a_{kj}\frac{\partial^2 v_{j\infty}(\tau)}{\partial\tau^2}-\frac{3!}{\left(1-v_{k\infty}\right)^4}\left(\frac{\partial v_{k\infty}(\tau)}{\partial\tau}\right)^3-\frac{4}{\left(1-v_{k\infty}\right)^3}\frac{\partial^2 v_{k\infty}(\tau)}{\partial\tau^2}\frac{\partial v_{k\infty}(\tau)}{\partial\tau}\end{aligned}$$

Again rewritten as

$$\frac{1}{\tau\left(1-v_{k\infty}\right)^{2}}\frac{\partial^{3}v_{k\infty}\left(\tau\right)}{\partial\tau^{3}}-\sum_{j=1}^{N}a_{kj}\frac{\partial^{3}v_{j\infty}\left(\tau\right)}{\partial\tau^{3}}=\frac{3}{\tau}\sum_{j=1}^{N}a_{kj}\frac{\partial^{2}v_{j\infty}\left(\tau\right)}{\partial\tau^{2}}-\frac{3!}{\tau\left(1-v_{k\infty}\right)^{4}}\left(\frac{\partial v_{k\infty}\left(\tau\right)}{\partial\tau}\right)^{3}$$
$$-\frac{6}{\tau\left(1-v_{k\infty}\right)^{3}}\frac{\partial^{2}v_{k\infty}\left(\tau\right)}{\partial\tau^{2}}\frac{\partial v_{k\infty}\left(\tau\right)}{\partial\tau}$$

leads to the matrix form

$$\mathcal{Q}\left(\frac{1}{\tau\left(1-v_{i\infty}\right)^{2}}\right)\frac{\partial^{3}V_{\infty}}{\partial\tau^{3}}=\frac{3}{\tau}A\frac{\partial^{2}V_{\infty}}{\partial\tau^{2}}-\frac{6}{\tau}\text{diag}\left(\frac{1}{\left(1-v_{k\infty}\right)^{4}}\right)\left(\frac{\partial V_{k\infty}\left(\tau\right)}{\partial\tau}\right)^{3}$$
$$-\frac{6}{\tau}\text{diag}\left(\frac{1}{\left(1-v_{k\infty}\right)^{3}}\right)\frac{\partial^{2}V_{k\infty}\left(\tau\right)}{\partial\tau^{2}}\frac{\partial V_{k\infty}\left(\tau\right)}{\partial\tau}$$

Introducing $A\frac{\partial^2 V_\infty}{\partial\tau^2}$ from (5) as

$$A\frac{\partial^{2}V_{\infty}}{\partial\tau^{2}}=\text{diag}\left(\frac{1}{\tau\left(1-v_{i\infty}\right)^{2}}\right)\frac{\partial^{2}V_{\infty}}{\partial\tau^{2}}-\frac{2}{\tau^{2}}\text{diag}\left(\frac{1}{\left(1-v_{i\infty}\right)^{2}}\right)\frac{\partial V_{\infty}}{\partial\tau}+$$
$$\frac{2}{\tau}\text{diag}\left(\frac{1}{\left(1-v_{k\infty}\right)^{3}}\right)\left(\frac{\partial V_{k\infty}\left(\tau\right)}{\partial\tau}\right)^{2}+\frac{2}{\tau^{3}}\text{diag}\left(\frac{1}{1-v_{i\infty}}\right)V_{\infty}$$

yields

$$\mathcal{Q}\left(\frac{1}{\tau\left(1-v_{i\infty}\right)^{2}}\right)\frac{\partial^{2}V_{\infty}}{\partial\tau^{2}}=R_{3}$$

where

$$R_{3}=\frac{6}{\tau^{4}}\frac{V_{\infty}}{1-V_{\infty}}-\frac{6}{\tau^{3}}\frac{\frac{\partial V_{\infty}}{\partial\tau}}{\left(1-V_{\infty}\right)^{2}}+\frac{3}{\tau^{2}}\frac{\frac{\partial^{2}V_{\infty}}{\partial\tau^{2}}}{\left(1-V_{\infty}\right)^{2}}+\frac{6}{\tau^{2}}\frac{\left(\frac{\partial V_{\infty}(\tau)}{\partial\tau}\right)^{2}}{\left(1-V_{\infty}\right)^{3}}$$
$$-\frac{6}{\tau}\frac{\left(\frac{\partial V_{\infty}(\tau)}{\partial\tau}\right)^{3}}{\left(1-V_{\infty}\right)^{4}}-\frac{6}{\tau}\frac{\frac{\partial^{2}V_{k\infty}(\tau)}{\partial\tau^{2}}\frac{\partial V_{k\infty}(\tau)}{\partial\tau}}{\left(1-V_{\infty}\right)^{3}}$$

The computation illustrates the general structure (1) and demonstrates the Theorem. □

From a numerical point of view, the non-linear NIMFA steady-state matrix equation (2) only needs be solved once for a particular value of τ so that the vector $V_\infty(\tau)$ is known, as well as the generalized Laplacian $\mathcal{Q}\left(\frac{1}{\tau(1-v_{i\infty})^2}\right)$. The Taylor expansion

$$V_{\infty}\left(\tau+\Delta\tau\right)=\sum_{m=0}^{\infty}\frac{\left(\Delta\tau\right)^{m}}{m!}\frac{\partial^{m}V_{\infty}\left(\tau\right)}{\partial\tau^{m}}\tag{6}$$

specifies the NIMFA infection probability vector $V_\infty(\tau+\Delta\tau)$ at another effective infection rate $\tau+\Delta\tau$, provided that the Taylor series converges at $\tau+\Delta\tau$. As mentioned earlier in [3], unfortunately, the convergence radius of the series in (6) is difficult to determine in general. The left-hand side positive definite matrix $\mathcal{Q}\left(\frac{1}{\tau(1-v_{i\infty})^2}\right)$ in (1) is the same for all orders $m\geq 1$ and can be inverted if a high precision and many terms in the Taylor series (6) are required.

The value of τ_u

We define $\chi_D(\tau) = \sum_{i=1}^{N} \sum_{j=1}^{i-1} 1_{f_{ij}(V(\tau),D)<0}$, then the larger χ_D is the higher the difference between the rankings of the infection probability at τ and the nodal degree is. As shown in S3, we plot $\chi_D(\tau)$ as a function of the average fraction y_∞ of infected nodes for ER and BA random graphs with the average degree $E[D] = 14$ as an example. We find that for both graphs $\chi_D \approx 0$ when the average fraction y_∞ of infected nodes is above 0.9, which suggests that we can employ the value of τ_u so that $y_\infty(\tau_u) = 0.9$. We have also done such tests on all the other networks in this paper and obtain the same conclusion. Hence, we employ the value τ_u, leading to $y_\infty(\tau_u) = 0.9$, for all networks in this paper.

This choice of 0.9, though not necessarily optimal, is supported by the following aspects. Practically, we would like to choose $y_\infty(\tau_u)$ as large as possible so that real-world prevalence levels are covered. Since real-world prevalence seldom reaches 0.9, $y_\infty(\tau_u) = 0.9$ is large enough. Also, we would like to choose $y_\infty(\tau_u)$ as large as possible so that $\chi(\tau_c^{(1)} + \epsilon, \tau_u)$ well counts the total number of crossings. Moreover, $y_\infty(\tau_u)$ should not be too large because the infection probability of the nodes are very close to each other when the prevalence is high, and the precision of numerical solution to compute the infection probability per node is not sufficient to distinguish nor to rank the nodes according to their infection probabilities. Furthermore, we observed that the crossing seldom happens when $y_\infty(\tau) > 0.9$ in all the networks generated by the two network models as well as in real-world networks. This is due to the fact that the number of crossings decreases as τ increases, as observed and discussed in the paper.

To compute the value of τ_u which leads to a high prevalence (0.9), we can employ the Laurent series of the steady-state infection probability [3, 1]:

$$v_{i\infty}(\tau) = 1 + \sum_{m=1}^{\infty} \eta_m(i)\,\tau^{-m} \tag{7}$$

where the coefficient $\eta_1(i) = -\frac{1}{d_i}$ and

$$\eta_2(i) = \frac{1}{d_i^2}\left(1 - \sum_{j=1}^{N} \frac{a_{ij}}{d_j}\right) \tag{8}$$

and for $m \geq 2$, the coefficients obey the recursion

$$\eta_{m+1}(i) = -\frac{1}{d_i}\eta_m(i)\left(1 - \sum_{j=1}^{N} \frac{a_{ij}}{d_j}\right) - \frac{1}{d_i}\sum_{k=2}^{m} \eta_{m+1-k}(i) \sum_{j=1}^{N} a_{ij}\eta_k(j)$$

Considering a large value of τ_u

$$v_{i\infty} = 1 - \frac{1}{\tau_u d_i} + O(\tau^{-2})$$

and

$$y_\infty = 1 - \frac{1}{\tau_u}\mathrm{E}[\frac{1}{D}] + O(\tau^{-2})$$

then, ignoring the second order condition $O(\tau^{-2})$,

$$\tau_u \approx \frac{1 - y_\infty}{\mathrm{E}[\frac{1}{D}]}$$

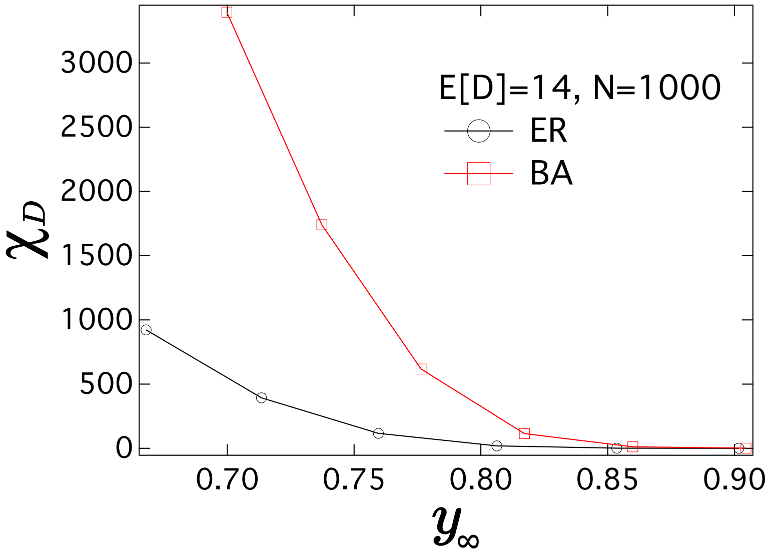

Figure S3: The plot of χ_D as a function of the average fraction of infected nodes. The results are averaged over 10 realizations

.

Real-world graphs

We use 6 connected and undirected graphs from real-world datasets. Some graphs are originally directed and may not be connected. We use the largest component of the unconnected graphs and change the directed graphs to undirected. The description of the 6 graphs are as follows:

1. GRQC: Arxiv GR-QC (General Relativity and Quantum Cosmology) collaboration network is from the e-print arXiv and covers scientific collaborations between authors papers submitted to General Relativity and Quantum Cosmology category. If an author i coauthored a paper with author j, there is link between i and j. The data covers papers in the period from January 1993 to April 2003.

2. NetSci: A coauthorship network of scientists working on network theory and experiment. The network was compiled from the bibliographies of two review articles on networks.

3. ODLIS: The network is based on the ODLIS: Online Dictionary of Library and Information Science (December 2000). The nodes are the terms in ODLIS and there is link between two terms if one is used to describe another one.

4. Roget: The network contains cross-references in Roget's Thesaurus, 1879. Each node of the graph corresponds to one of the categories in the 1879 edition of Peter Mark Roget's Thesaurus of English Words and Phrases. There is a link between two categories if one is the reference of the other. (See http://vlado.fmf.uni-lj.si/pub/networks/data/dic/roget/Roget.htm)

5. Power: The network represents the topology of the Western States Power Grid of the United States.

6. Yeast: The protein-protein interaction network in budding yeast. There is link between protein i and protein j if they have the interaction.

In Table S3, we list the size N, the average degree $E[D]$, the degree variance $Var[D]$ and the normalized degree variance $Var^*[D]$ of the 6 graphs.

Table S3: The real-world graph used in this paper.

	GRQC	NetSci	ODLIS	Roget	Power	Yeast
N	4158	379	2898	994	4941	2224
$E[D]$	6.46	4.82	11.30	7.32	2.67	5.94
$Var[D]$	74.42	15.46	679.61	23.66	3.21	63.70

The comparison between NIFMA and the continuous-time simulation

We compare the number of crossings obtained by NIMFA and the simulations of the exact SIS model. We show two examples of the comparison in Fig. S4. Because the NIMFA epidemic threshold $\tau_c^{(1)}$ is actually the lower bound of the real epidemic threshold, i.e. $\tau_c^{(1)} < \tau_c$, and to determine the value of $\alpha_c = \frac{\tau_c}{\tau_c^{(1)}} > 1$ for different topology is difficult, we start the comparison from $\alpha = 2$ (attempting to exclude the crossings near the epidemic threshold). Fig. S4 shows that the results of the simulation and NIMFA agree with each other quite well for both networks when α is not large. When α is large, i.e. the infection probability of each node is high and close to each other, there might be some crossings caused by the limited precision of the numerical NIMFA solution or the simulations. Because the precision of the numerical solution is higher than that of the simulation of the exact SIS model, the number χ of crossings obtained from the simulation tends to be larger than that from NIMFA if the effective infection rate α is large.

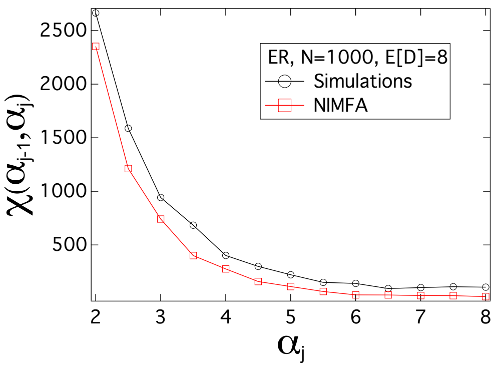

(a)

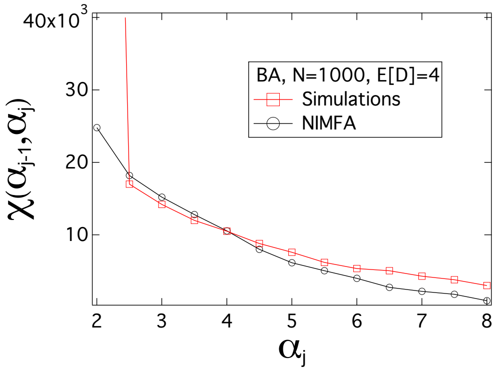

(b)

Figure S4: The comparison of the number χ of crossings between NIMFA and the simulation of the exact SIS model for (a) an ER random graph with $N = 1000$ and $E[D] = 8$; (b) a BA random graph with $N = 1000$ and $E[D] = 4$. The linear sampling is employed with the step $\Delta\alpha = 0.5$.

The normalized standard deviation σ^* of the steady-state infection probability

ER random graphs with $N = 1000$

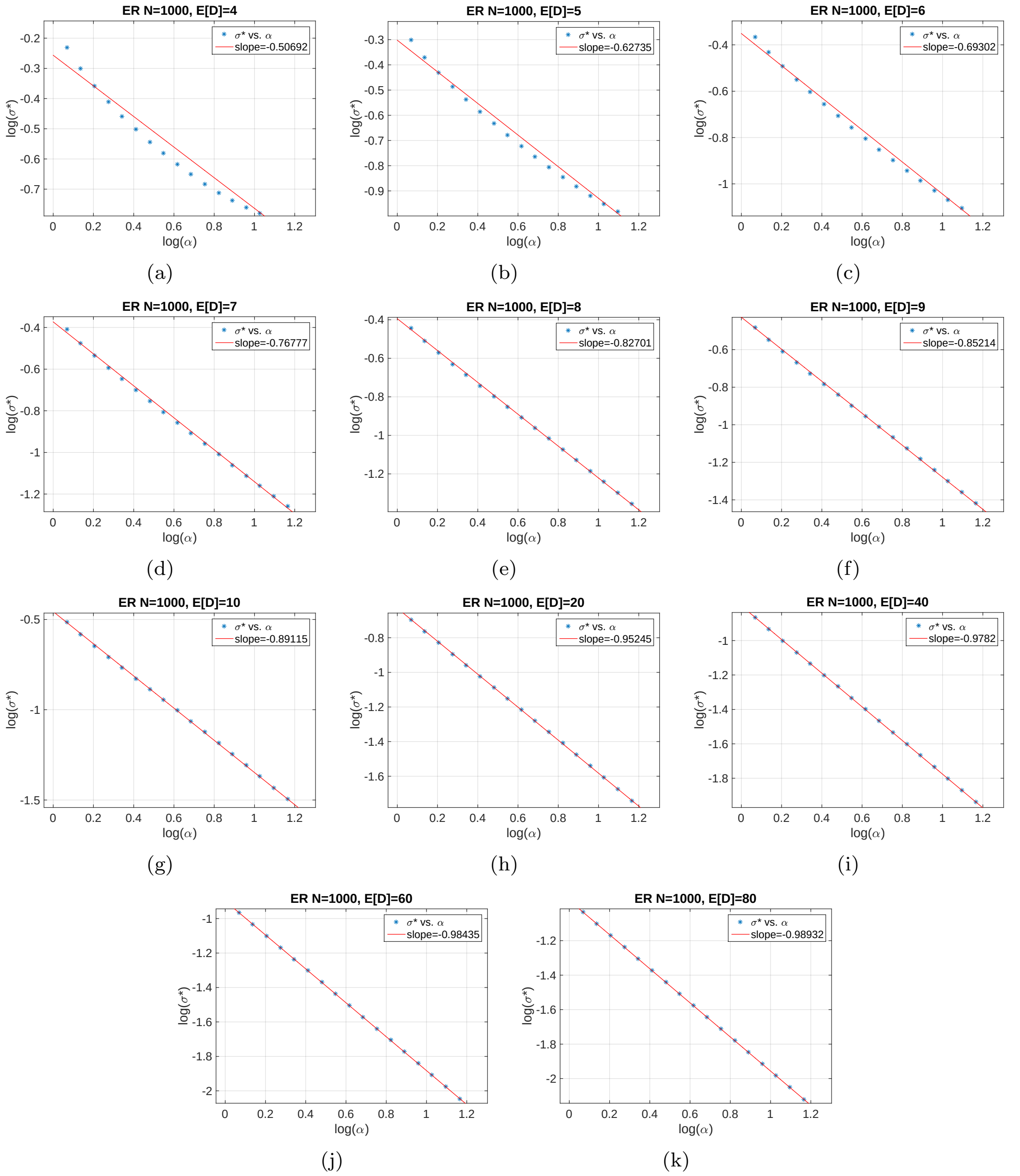

Figure S5: σ^* as a function of α for ER random graphs and the corresponding fitting curve.

BA random graphs with $N = 1000$

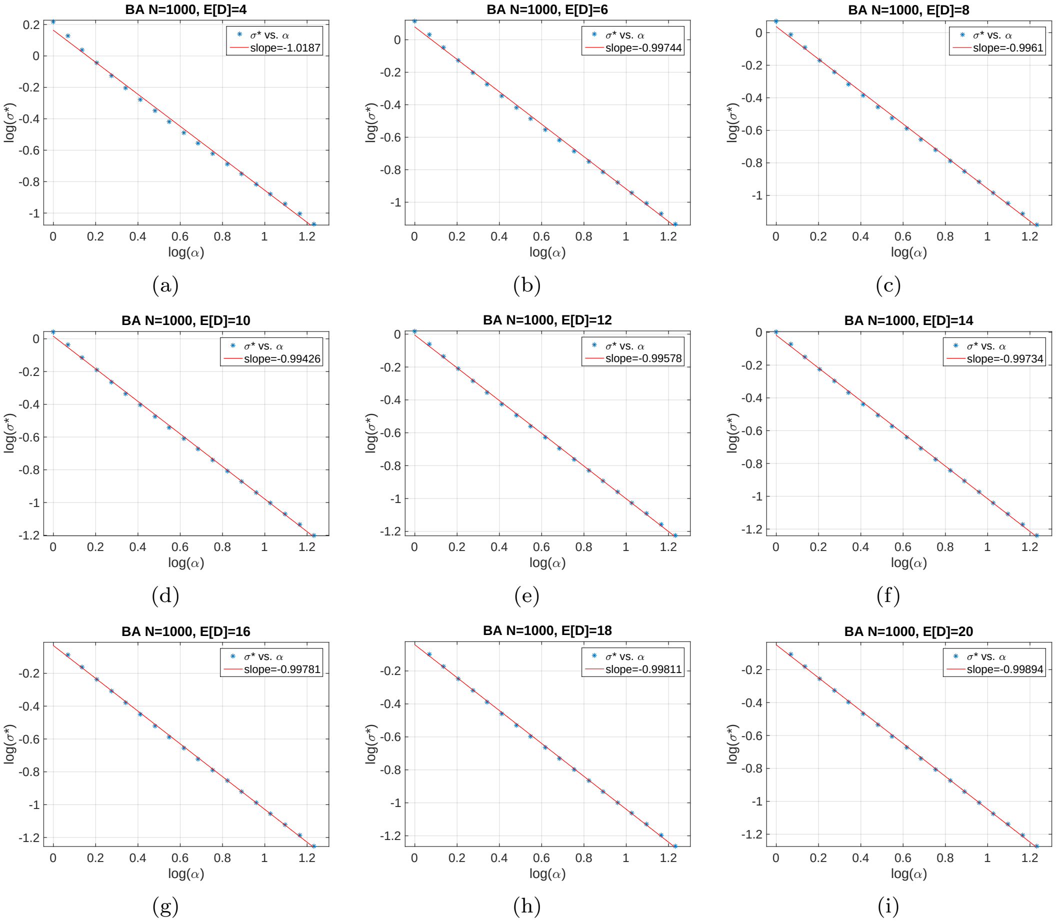

Figure S6: σ^* as a function of α for BA random graphs and the corresponding fitting curve.

σ^* as a function of τ

Here we show the relationship between σ^* and τ when $\tau \gg 1$.

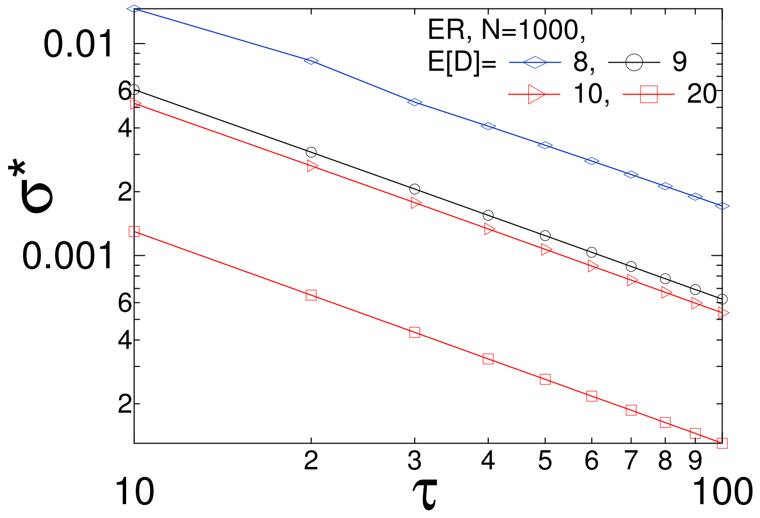

(a)

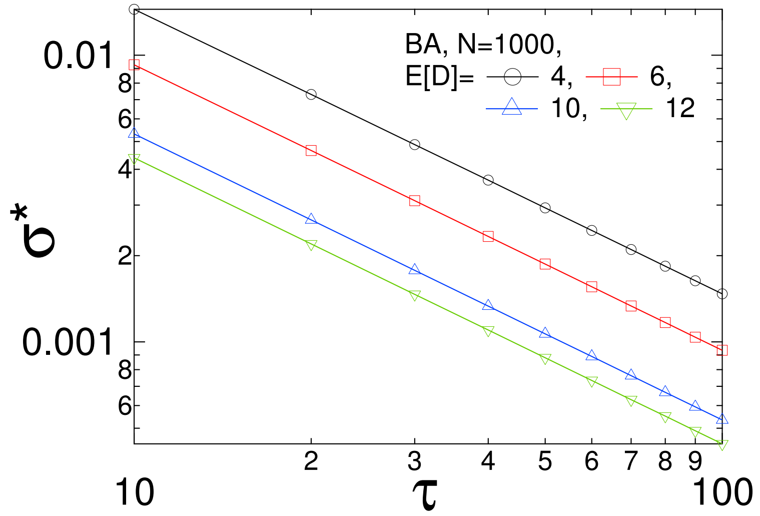

(b)

Figure S7: σ^* as a function of τ for (a) ER random graphs with the size $N = 1000$ and (b) BA random graphs with the size $N = 1000$.

The Spearman rank correlation ρ as a function of α

In Fig. S8, we choose two graphs, an ER random graph with $\mathrm{E}[D] = 8$ and a BA random graph with $\mathrm{E}[D] = 4$, as the example. We plot Spearman Rank Correlation between $V_\infty(\tau_c^{(1)} + \epsilon)$ and $V_\infty(\alpha\tau_c^{(1)})$. We find that the rank correlation decreases fast when the effective infection rate is small. Moreover, there tend to be a few nodes drastically changing ranks in BA random graphs but not in ER graphs.

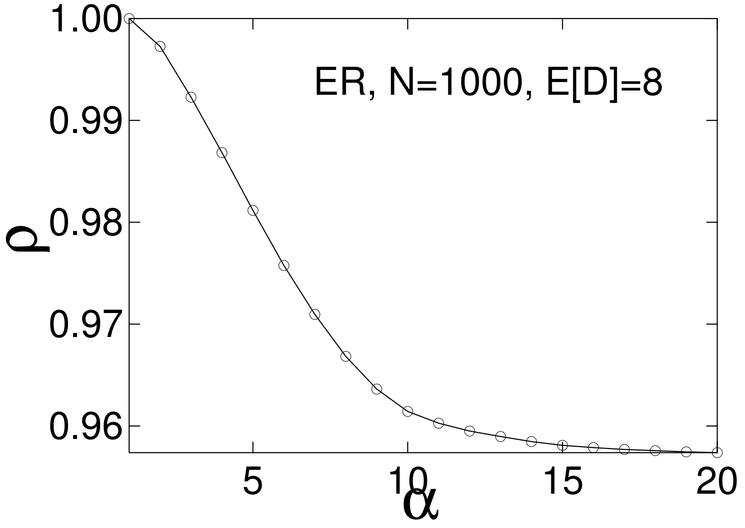

(a)

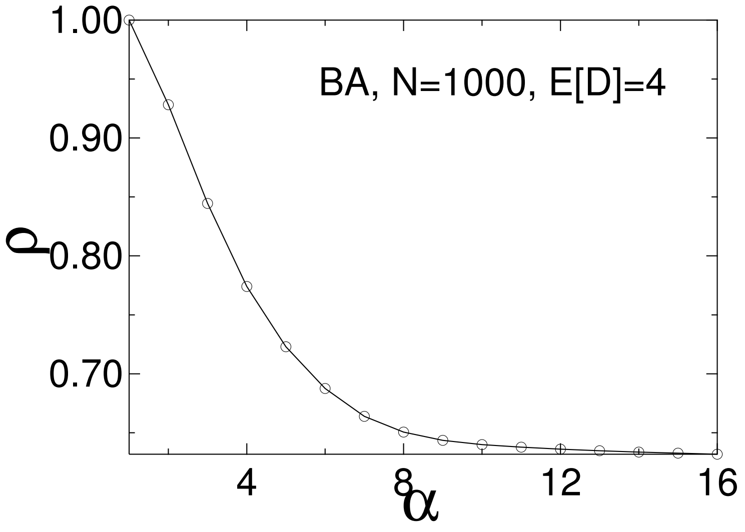

(b)

Figure S8: The Spearman rank correlation between $V_\infty(\tau_c^{(1)} + \epsilon)$ and $V_\infty(\alpha\tau_c^{(1)})$ as a function of α for (a) ER random graphs with the size $N = 1000$ and the average degree $\mathrm{E}[D] = 8$ and (b) BA random graphs with the size $N = 1000$ and the average degree $\mathrm{E}[D] = 4$.

References

[1] Piet Van Mieghem. *Performance analysis of communications networks and systems.* Cambridge University Press, 2014.

[2] Piet Van Mieghem and Jasmina Omic. In-homogeneous virus spread in networks. *arXiv preprint arXiv:1306.2588*, 2013.

[3] Piet Van Mieghem. The viral conductance of a network. *Computer Communications*, 35(12):1494–1506, 2012.
